# Periodontitis and Gestational Diabetes Mellitus: Evidence for a Potential Biological Link

**DOI:** 10.3290/j.ohpd.c_2840

**Published:** 2026-11-11

**Authors:** Linxin Jiang, Simin Li, Meng Luo, Shaonan Hu, Zhihao Liao, Qian Liu, Xin Deng, Daniel R Reissmann, Deborah Kreher, Gerhard Schmalz, Xianda Hu

**Affiliations:** a Linxin Jiang* Doctoral Researcher, Department of Prosthodontics, University of Leipzig, 04103 Leipzig, Germany; involved in the conceptualisation of the study, data curation, formal analysis, funding acquisition, visualisation, and writing of the original draft; b Simin Li* Postdoctoral Researcher, Stomatological Hospital, School of Stomatology, Southern Medical University, Guangzhou 510280, Guangdong, China; involved in the investigation, methodology, software, and writing of the original draft; c Meng Luo Physician, Department of Anesthesiology, Sichuan Province Orthopedic Hospital, Chengdu, Sichuan 610041, China; involved in the formal analysis, methodology, and validation; d Shaonan Hu Postdoctoral Researcher, Stomatological Hospital, School of Stomatology, Southern Medical University, Guangzhou 510280, Guangdong, China; involved in the data curation and formal analysis; e Zhihao Liao Doctoral Researcher, Stomatological Hospital, School of Stomatology, Southern Medical University, Guangzhou 510280, Guangdong, China; involved in the investigation and methodology; f Qian Liu Doctoral Researcher, Department of Prosthetic Dentistry, Center for Dental Medicine, Medical Center – University of Freiburg, Faculty of Medicine, University of Freiburg, 79106 Freiburg, Germany; Doctoral Researcher, GERN Research Center for Tissue Replacement, Regeneration & Neogenesis, Department of Prosthetic Dentistry, Medical Center – University of Freiburg, Faculty of Medicine, University of Freiburg, 79108 Freiburg, Germany; involved in the visualisation; g Xin Deng Postdoctoral Researcher, Stomatological Hospital, School of Stomatology, Southern Medical University, Guangzhou 510280, Guangdong, China; involved with the software; h Daniel R Reissmann Professor, Department of Prosthodontics, University of Leipzig, 04103 Leipzig, Germany; involved in the conceptualisation of the study, project administration, supervision, and reviewing and editing of the manuscript; i Deborah Kreher Physician, Department of Conservative Dentistry and Periodontology, Brandenburg Medical School Theodor Fontane (MHB), 14770 Brandenburg/Havel, Germany; involved with the methodology; j Gerhard Schmalz# Professor, Department of Conservative Dentistry and Periodontology, Brandenburg Medical School Theodor Fontane (MHB), 14770 Brandenburg/Havel, Germany; involved in the conceptualisation of the study, project administration, supervision, and reviewing and editing of the manuscript; k Xianda Hu Department Manager, Laboratory of Molecular Cell Biology, Beijing Tibetan Hospital, China Tibetology Research Center, Chaoyang, Beijing 100029, China; involved in the conceptualisation of the study, project administration, supervision, and reviewing and editing of the manuscrip *Equal contribution. #Senior author.

**Keywords:** bioinformatics analysis, experimental validation, gestational diabetes mellitus, Mendelian randomisation, periodontitis

## Abstract

**Purpose:**

This study aimed to investigate the potential association between gestational diabetes mellitus (GDM) and periodontitis (PD) and to explore possible biological mechanisms underlying this link.

**Materials and methods:**

In the first part of this study, a two-sample Mendelian randomisation (MR) analysis was conducted to evaluate the potential effect of PD on the risk of GDM, and vice versa. In the second part, transcriptomic data and network-based bioinformatics analyses were integrated, including differential expression analysis and protein-protein interaction (PPI) network construction, to screen for common differentially expressed genes and significantly enriched biological pathways. In the third part, a simplified in vitro β-cell challenge system was established using the human pancreatic β-cell line EndoC-βH1. Cells were exposed to high-glucose and progesterone conditions with or without Porphyromonas gingivalis to examine inflammatory and oxidative stress responses. Differences in the expression of MR-genes, inflammatory cytokines, and reactive oxidative species were evaluated. All analyses were performed using R version 4.2.2 (R CoreTeam, Vienna, Austria).

**Results:**

The forward MR analysis showed evidence consistent with a modest positive association of genetically predicted PD with GDM risk (OR = 1.14, *P* = 0.006), whereas the reverse analysis did not provide evidence for an association of genetically predicted GDM with PD risk (OR = 1.08, *P* = 0.222). Sensitivity analyses supported the robustness of the MR results. Transcriptomic bioinformatic analyses identified multiple key genes that were dysregulated in both PD and GDM, including inflammatory mediators (IL1RN and TGFB1) and metabolic regulators (AKT1, NOS3, and TCF7L2). Functional enrichment revealed that the overlapping genes were mainly involved in critical biological processes like apoptotic signalling, cell adhesion, and inflammatory responses. The PPI network suggested a synergistic role of PD and GDM in inflammation and metabolic regulation. Compared with the GDM model, β-cells in the GDM+PD model exhibited significantly enhanced inflammatory responses and oxidative stress. Additionally, the MR-genes showed significant expression changes in the GDM+PD model: HSPBAP1, APBA1, and SHC4 were upregulated, whereas CDH1, NDUFAF6, and DGKB were downregulated.

**Conclusion:**

These findings provide evidence consistent with a potential biological link between PD and GDM, in which periodontal infection-related inflammatory responses and oxidative stress may aggravate β-cell stress under GDM-like conditions.

Gestational diabetes mellitus (GDM) is defined as chronic hyperglycaemia identified during pregnancy in women who were not previously diagnosed with diabetes.^30^ It is one of the most common complications of pregnancy and can lead to adverse outcomes such as preterm birth, macrosomia, neonatal jaundice, and neonatal respiratory distress syndrome.^41^ The hyperglycaemia associated with GDM does not always resolve after childbirth. Women with a history of GDM have around an eightfold higher risk of developing type 2 diabetes, and up to one-third will progress to diabetes within 15 years.^9^ In recent years, the global prevalence of GDM has been increasing. Due to differences in regions and screening criteria, its prevalence ranges from 5% to 20% across different countries or regions,^2,13,27
^ with a global age-standardised prevalence of approximately 14%.^37^ Key risk factors for GDM include advanced maternal age, obesity, and dietary and lifestyle factors. However, intervention trials targeting GDM have not yielded significantly improved outcomes.^42^


Periodontitis (PD) is a chronic inflammatory disease caused by dysbiosis of the oral microbiota. It leads to progressive destruction of the periodontal supporting tissues, manifested by irreversible alveolar bone resorption and possible tooth loss.^22^ In contrast to GDM, severe periodontitis affected around 1.1 billion people worldwide in 2019, and its prevalence continues to increase.^5^ Periodontal pathogens and their virulence factors are not only confined to the oral cavity, but can also enter systemic circulation through the ulcerated pocket epithelium at diseased sites, inducing a sustained low-grade inflammatory state throughout the body.^3^ Such chronic systemic inflammation has been linked to an elevated risk of various systemic conditions, including cardiovascular disease, Alzheimer’s disease, respiratory illnesses, and adverse pregnancy outcomes.^35^ Meanwhile, the hormonal changes during pregnancy (such as increased estrogen and progesterone) can disrupt the balance of the oral microbiome, thereby raising the risk of PD and even adverse pregnancy outcomes.^20^ In summary, GDM and PD are common pregnancy-related metabolic and infectious conditions, respectively, and they may be epidemiologically associated, potentially influencing each other through inflammation and immune pathways.

Given the well-established bidirectional relationship between periodontitis and diabetes mellitus,^14^ a potential two-way relationship between GDM and PD has also drawn attention. On one hand, PD can adversely affect glycaemic control,^4^ and pregnant women with PD have around a 66% higher risk of developing GDM.^1^ On the other hand, the hyperglycaemic milieu of pregnancy can in turn exacerbate periodontal health problems through multiple mechanisms. A high-glucose environment aggravates inflammatory damage and impairs tissue repair,^39^ making it easier for periodontal pathogens to proliferate and thereby increasing the occurrence and severity of PD.^28^ In addition, the immune tolerance and hormonal environment of pregnancy (such as elevated levels of estrogen and relaxin) may render the host more susceptible to periodontal pathogens, amplifying the detrimental effect of PD on glucose metabolism.^7,38
^ This implies that PD and GDM could potentially promote each other via an inflammation-mediated vicious cycle. Overall, the above evidence supports the notion that periodontitis and GDM might share an interrelated pathophysiological basis.

However, current research focusing on PD and GDM is rare, and most of the existing studies are observational cross-sectional or case-control designs. Such designs are prone to confounding factors and reverse causation, making it difficult to establish a clear causal relationship between the two diseases.^6,19,23
^ Moreover, the biological mechanisms linking PD and GDM remain poorly understood. Most previous studies have focused on the impact of periodontitis on other pregnancy complications like preterm birth and preeclampsia,^19,21
^ or have examined the oral microbiome and specific periodontal pathogens in pregnancy.^8,40
^ Few studies have directly investigated the causal association between PD and GDM, and it is still unclear which inflammatory or metabolic pathways might mediate their potential connection.

Therefore, based on this background, the present authors designed a multi-stage study to clarify the causal association and underlying mechanisms between PD and GDM. To make the multistage design easier to follow, the present study was organised as a sequential evidence framework. First, MR analysis was used to explore whether genetic evidence supports a potential relationship between PD and GDM. Second, transcriptomic and network analyses were employed to identify candidate genes and pathways that might connect the two conditions. Third, a simplified in vitro β-cell challenge system was used to examine whether periodontal bacterial exposure could aggravate inflammatory and oxidative responses under GDM-like conditions. The aim of this study was to investigate the potential relationship between PD and GDM using genetic, transcriptomic, and cellular evidence, and to explore candidate biological pathways that may inform future interdisciplinary research.

## Materials and methods

### Study Design and Data Resource

This study was designed as a sequential, multistage investigation rather than three independent analyses. The MR analysis was used as an initial genetic epidemiological approach to evaluate the direction of the PD-GDM association. The bioinformatics analysis was then used to screen candidate genes and pathways that may explain this association. Finally, the cell experiment was used as a simplified in vitro challenge system to examine whether exposure to a periodontal pathogen could enhance inflammatory and oxidative stress responses in pancreatic β-cells under GDM-like conditions.

In part one, the two-sample MR was used to evaluate the causal relationship between PD and GDM. Summary-level genome-wide association study (GWAS) data for PD and GDM were obtained from the Integrative Epidemiology Unit OpenGWAS database (IEU OpenGWAS) (https://gwas.mrcieu.ac.uk/). The PD GWAS dataset (ID: finn-b-K11_PERIODON_CHRON) included 3,046 PD cases and 195,395 controls of European ancestry, and the GDM GWAS dataset (ID: finn-b-GEST_DIABETES) included 5,687 GDM cases and 117,892 controls of European ancestry.

In part two, integrative bioinformatics analyses were performed to identify MR-related genes and potential molecular mechanisms. Transcriptomic datasets for PD and GDM were obtained from the National Center for Biotechnology Information (NCBI) Gene Expression Omnibus (GEO) database (http://www.ncbi.nlm.nih.gov/geo/). The PD dataset GSE61490 contains gene expression profiles from 5 PD patients and 5 healthy controls, and the GDM dataset GSE203346 contains profiles from 21 GDM patients and 22 healthy controls. Additionally, disease-associated genes were retrieved from the DisGeNET database (https://www.disgenet.org/home/), obtaining a list of 649 PD-related genes and 682 GDM-related genes.

In part three, cellular experiments were carried out using the human pancreatic β-cell line EndoC-βH1 as a simplified in vitro β-cell challenge system under GDM-like conditions with or without *Porphyromonas gingivalis* (Pg) exposure. High-glucose medium plus progesterone was used to mimic the GDM environment, and co-culture with the periodontopathogenic bacterium Pg was used to simulate concurrent PD. This experimental setup was used to provide exploratory biological evidence for the potential link between PD-related bacterial exposure and β-cell inflammatory responses under GDM-like conditions.

### MR Analysis

#### Instrumental variable (IV) selection

MR analysis relies on instrumental variables (IVs) that satisfy three key assumptions: the association assumption, the independence assumption, and the exclusion restriction assumption (Fig 1). Single-nucleotide polymorphisms (SNPs) were selected as IVs for the exposure (PD or GDM) based on the following criteria: SNPs associated with the exposure were selected using a significance threshold of *P* < 5 ×10^-6^. This relatively relaxed threshold was adopted because few variants reached conventional genome-wide significance for the available PD and GDM datasets; therefore, the MR analysis should be regarded as exploratory. To ensure independence among instruments, linkage disequilibrium clumping was performed using an R^2^ threshold of 0.001 within a 10,000-kb window. SNPs with an F statistic < 10 were excluded to reduce weak instrument bias. The F statistic was calculated as F = β^2^/SE^2^ for each SNP, where β and SE represent the SNP-exposure association estimate and its standard error, respectively. The variance explained by each instrument was estimated using R^2^ = 2 × EAF × (1 − EAF) × β^2^, where EAF represents the effect allele frequency.

**Fig 1 Fig1:**
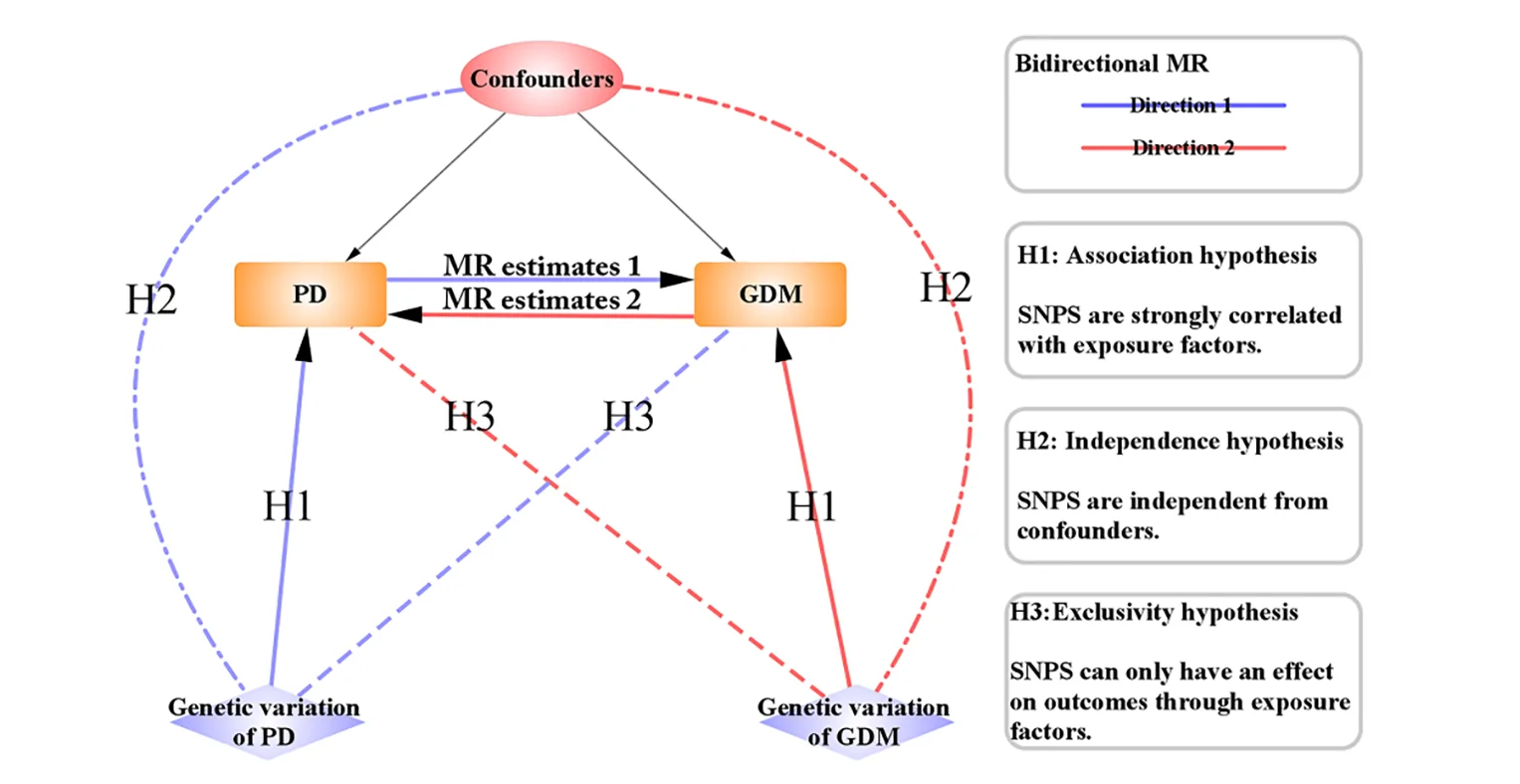
Framework of the MR analysis for PD and GDM. H1–H3 represent the three fundamental MR assumptions, including the relevance assumption (H1), the independence assumption (H2), and the exclusion restriction assumption (H3).

#### Data harmonisation

Exposure and outcome datasets were harmonised to ensure that the SNP exposure and SNP outcome effects corresponded to the same effect allele. During harmonisation, alleles were aligned across exposure and outcome datasets, and ambiguous palindromic variants were checked using allele frequency information where available. Palindromic SNPs with intermediate allele frequencies or ambiguous strand orientation were excluded to avoid potential strand misalignment. SNPs unavailable in the outcome dataset were excluded if they were unavailable in the outcome dataset.

#### MR estimates

Several complementary MR methods were used to estimate the potential effect of the exposure on the outcome, including inverse-variance weighted (IVW), MR-Egger regression, weighted median, simple mode, and weighted mode methods. The IVW method was considered the primary analysis because it provides the most statistically efficient estimate when all instruments are valid or when horizontal pleiotropy is balanced. Complementary methods, including MR-Egger regression, weighted median, simple mode, and weighted mode, were used to assess the consistency of the findings under different assumptions about instrument validity. The weighted median method can provide a consistent estimate when at least 50% of the weight comes from valid instruments, whereas MR-Egger regression allows for directional pleiotropy but has lower statistical power, especially when the number of instruments is small. In the absence of evidence for pleiotropy, the IVW estimate was taken as the main MR result, even if some heterogeneity was present.

#### Sensitivity analyses

Several sensitivity analyses assessed the robustness of the MR findings:

A Cochran Q statistic was calculated for both the IVW and MR-Egger models to test for heterogeneity among the SNPs, with *P* < 0.05 indicating significant heterogeneity. A MR-Egger intercept test was used to detect horizontal pleiotropy: a non-zero intercept with *P* < 0.05 would indicate significant pleiotropy, meaning that the IVs have directional effects on the outcome independent of the exposure. A leave-one-out analysis was conducted by removing each SNP at a time and re-estimating the effect to determine whether any single SNP disproportionately influenced the overall MR estimate. If the overall effect estimate changed markedly upon exclusion of a particular SNP, the result would be considered sensitive to that SNP. A funnel plot of the individual SNP effects for symmetry was examined: an asymmetrical funnel plot could suggest the presence of directional pleiotropy or other biases. 

### Bioinformatics Analysis

The bioinformatics analyses were exploratory and were intended to prioritise candidate genes and pathways for subsequent interpretation rather than to establish definitive mechanisms.

#### MR-related candidate genes

From the MR analysis results, SNPs contributing to the MR association were annotated using the dbSNP database and mapped to nearby or annotated genes based on the hg19 reference genome. These genes were defined as MR-related candidate genes for exploratory interpretation only. This mapping does not establish causal genes or direct biological mechanisms, particularly because several variants were intronic or intergenic.

#### PD-differentially expressed genes (DEGs) and GDM-DEGs

The lists of genes associated with PD and GDM were obtained from the DisGeNET database, comprising 649 PD-related genes and 682 GDM-related genes. The intersection of these two lists yielded a set of genes common to both PD and GDM. The expression data were extracted for these overlapping genes from the PD and GDM transcriptomic datasets and performed differential expression analysis (cases vs controls) in each dataset. Given the exploratory purpose of this analysis and the small sample size of the PD transcriptomic dataset, genes meeting the criteria of nominal *P* < 0.05 and |log2(FC)| > 0.07 were retained as candidate DEGs for downstream analyses. This permissive threshold was used to capture modest but potentially coordinated expression changes. Multiple-testing correction was not applied in the primary screening; therefore, these results should be interpreted as hypothesis-generating. These DEGs were labelled as PD-DEGs and GDM-DEGs, respectively. The differences were then analysed in expression distributions of these DEGs between case and control groups and the correlations among the DEGs were assessed within each disease context.

#### Functional enrichment analysis

The PD-DEGs, GDM-DEGs, and MR-genes were combined to form a target gene set (crosstalk genes). To gain insight into the biological roles of these genes, functional enrichment analysis was performed. Gene Ontology (GO) enrichment analysis for biological process terms and Kyoto Encyclopedia of Genes and Genomes (KEGG) pathway enrichment analysis were carried out to identify the GO terms and pathways significantly associated with the crosstalk gene set.

#### Protein-protein interaction (PPI) network construction

The known PPI was retrieved from the Human Protein Reference Database (HPRD) (http://www.hprd.org/) and BioGRID (http://thebiogrid.org/) database and these interaction pairs were merged. To explore how the MR-genes connect with other genes, the PPI data were filtered to identify interactions involving any of the MR-genes. The direct interacting partners of MR-genes were labelled as the Target1 gene set. The interaction partners of these Target1 genes (second–degree interactors) were then identified and labelled as the Target2 gene set. From the Target2 genes, those that overlapped with the present authors’ PD-DEGs or GDM-DEGs were extracted. Finally, a network was constructed that included the MR-genes, Target1 and Target2 genes, and the PD- and GDM-specific DEGs identified (MR-gene-Target1-Target2 network). This PPI network was analysed and visualised to understand the potential interactions linking MR-genes with key dysregulated genes in PD and GDM.

#### Pathway–gene crosstalk analysis

From the KEGG pathway enrichment results, the pathways that were significantly associated with the crosstalk gene set were identified. For each pathway, the list of member genes from the KEGG database (https://www.kegg.jp/) was obtained. The crosstalk genes were then mapped onto these pathways, identifying pathway–gene pairs for each significant pathway. In these pathway–gene relationships, genes belonging to the crosstalk set are highlighted, and bridging genes are annotated. This analysis enabled the observation of how the target gene set is functionally connected to key biological pathways.

### Exploratory in Vitro Assessment

#### Experimental design

The experiment investigated whether exposure to Pg could aggravate inflammatory cytokine expression, MR-related candidate gene expression, and oxidative stress in EndoC-βH1 cells under high-glucose and progesterone conditions. EndoC-βH1 cells were exposed to high-glucose RPMI-1640 medium containing 4.5 g/l glucose and supplemented with progesterone to establish a GDM-like glucotoxic and hormonal condition. For the bacterial challenge condition, cells were additionally exposed to Pg as a simplified periodontal bacterial stimulus.^15,32
^ These conditions were used as a simplified in vitro β-cell challenge system and should not be interpreted as complete biological models of GDM or periodontitis. No separate cell viability or cytotoxicity assay was performed under the same exposure conditions. Two groups of EndoC-βH1 cells were established:

GDM group: Cells were cultured in high-glucose medium and treated with progesterone to mimic gestational diabetes conditions.GDM+PD group: Cells under high-glucose and progesterone conditions were co-cultured with the periodontitis-associated bacterium Pg to simulate the combined environment of GDM with periodontitis. 

#### Cell lines and cell cultures

The human pancreatic β-cell line (EndoC-βH1) was used. EndoC-βH1 cells were maintained in RPMI-1640 culture medium (HyClone; Cytiva, Marlborough, MA, USA; cat. no. SH30809.01B) containing high glucose (4.5 g/l) at 37°C in a humidified incubator with 5% CO_2_.

#### Quantitative real-time polymerase chain reaction (qRT-PCR) 

Total RNA was extracted from cells using TRIzol reagent (Invitrogen, Carlsbad, CA, USA). RNA purity and concentration were assessed with a BioPhotometer plus spectrophotometer (Eppendorf, Hamburg, Germany), and samples were confirmed to be free of protein contamination. The integrity of the RNA was verified by 1.5% agarose gel electrophoresis (run at 160 V for 15 minutes) to visualise the ribosomal RNA bands. Next, 1 μg total RNA was reverse-transcribed into cDNA using the EasyScript First-Strand cDNA Synthesis SuperMix kit (Vazyme, Nanjing, China). The resulting cDNA was used as a template for quantitative PCR, which was performed using a 2× SYBR Green qPCR SuperMix (Vazyme) on an ABI PRISM 7500 sequence detection system (Applied Biosystems, Waltham, MA, USA). Each sample was run in triplicate, and glyceraldehyde-3-phosphate dehydrogenase (GAPDH) was used as the housekeeping gene for internal normalisation. Relative gene expression levels were calculated using the 2^^-ΔΔCt^ method.

#### Western blotting (WB)

Protein expression levels were evaluated by WB. Cells were lysed to extract total protein, and the protein concentration of each sample was quantified. Equal amounts of protein from each sample were separated by SDS-PAGE and transferred onto PVDF membranes. The membranes were then incubated with primary antibodies against the target proteins and against the loading control protein GAPDH, followed by appropriate horseradish peroxidase (HRP)–conjugated secondary antibodies. Bound antibodies were detected using an enhanced chemiluminescence substrate, and the signal was captured using a chemiluminescent imaging system. Band intensities were analysed with ImageJ software (National Institutes of Health, Bethesda, MD, USA). Target protein signals were normalised to GAPDH and expressed as relative protein levels. Each experiment was performed in three independent replicates.

#### ROS detection

Intracellular ROS were detected using a fluorescence-based ROS assay kit (Beyotime, Shanghai, China; product no. S0033). Cells from each group were incubated with the fluorescent ROS probe according to the manufacturer’s protocol, and the fluorescence intensity was observed under a microscope and measured to assess the level of ROS generation in the cells.

### Statistical Analysis

All data processing and statistical analyses were carried out using R software (version 4.2.2). For comparisons of continuous variables, a Wilcoxon rank-sum test was used for non-normally distributed data and a Student *t* test was used for normally distributed data. Pearson correlation coefficients were calculated for correlation analyses. The PPI network was constructed and visualised using Cytoscape (version 3.9.1). A two-tailed *P* value < 0.05 was considered statistically significant for all analyses.

## Results

### MR Analysis 

#### MR estimates

A total of 12 SNPs were included in the forward MR analysis of PD on GDM risk, and 16 SNPs were included in the reverse MR analysis of GDM on PD risk. The inverse-variance weighted (IVW) method found evidence consistent with a modest positive association between genetically predicted PD and GDM risk (OR = 1.14, *P* = 0.006) (Table 1). In the reverse analysis, there was no clear evidence supporting an association between genetically predicted GDM and PD risk (OR = 1.08, *P* = 0.222). In the forward analysis, all other MR methods yielded effect estimates in the same direction as the IVW model (Fig 2a), and the overall combined effect estimate was positive (Fig 2b), indicating consistency across methods.

**Table 1 Table1:** MR estimate between PD and GDM

Exposure	Outcome	Method	Number of SNPs	SE	OR	*P* value
PD	GDM	MR Egger	12	0.118	1.13	0.323
PD	GDM	Weighted median	12	0.138	1.15	0.032
PD	GDM	IVW	12	0.128	1.14	0.006
PD	GDM	Simple mode	12	0.143	1.15	0.220
PD	GDM	Weighted mode	12	0.145	1.16	0.209
GDM	PD	MR Egger	16	−0.116	0.89	0.505
GDM	PD	Weighted median	16	0.077	1.08	0.365
GDM	PD	IVW	16	0.073	1.08	0.222
GDM	PD	Simple mode	16	0.117	1.12	0.453
GDM	PD	Weighted mode	16	0.097	1.10	0.487
OR, odds ratio; SE, standard error.						

**Fig 2a to d Fig2atod:**
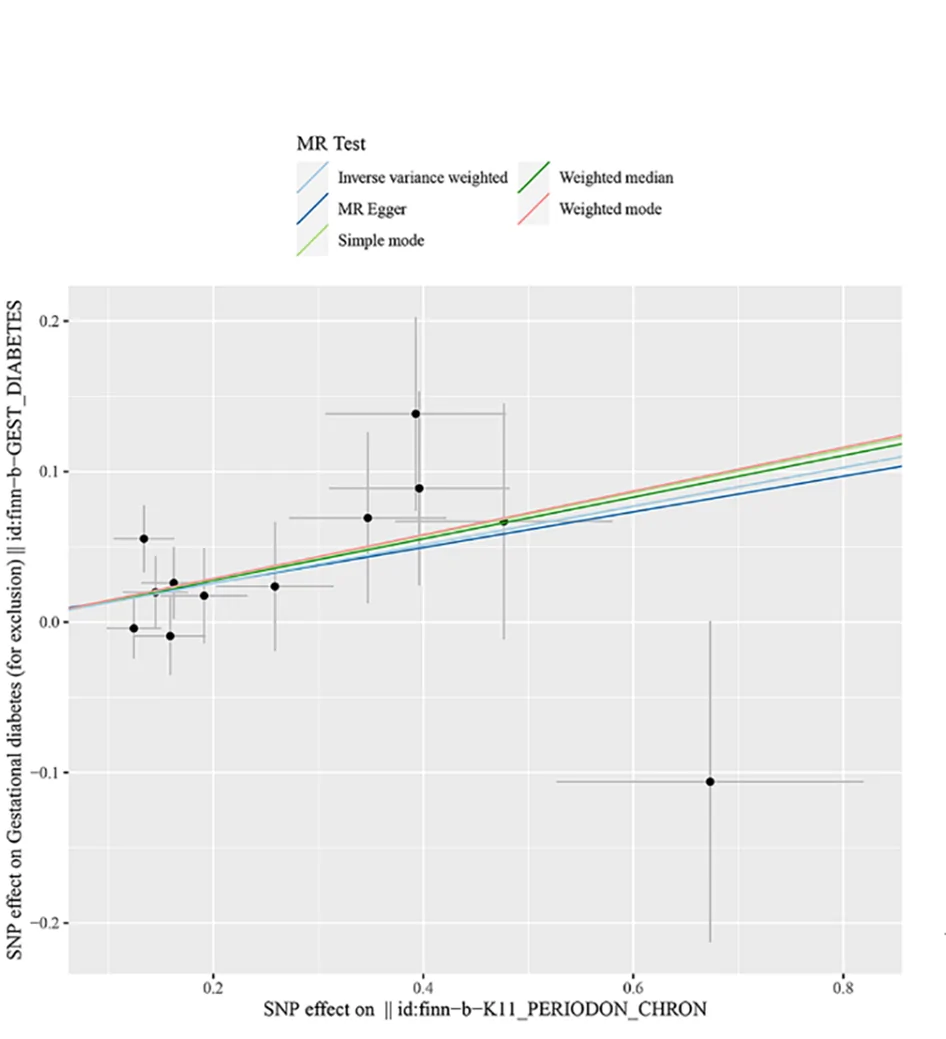

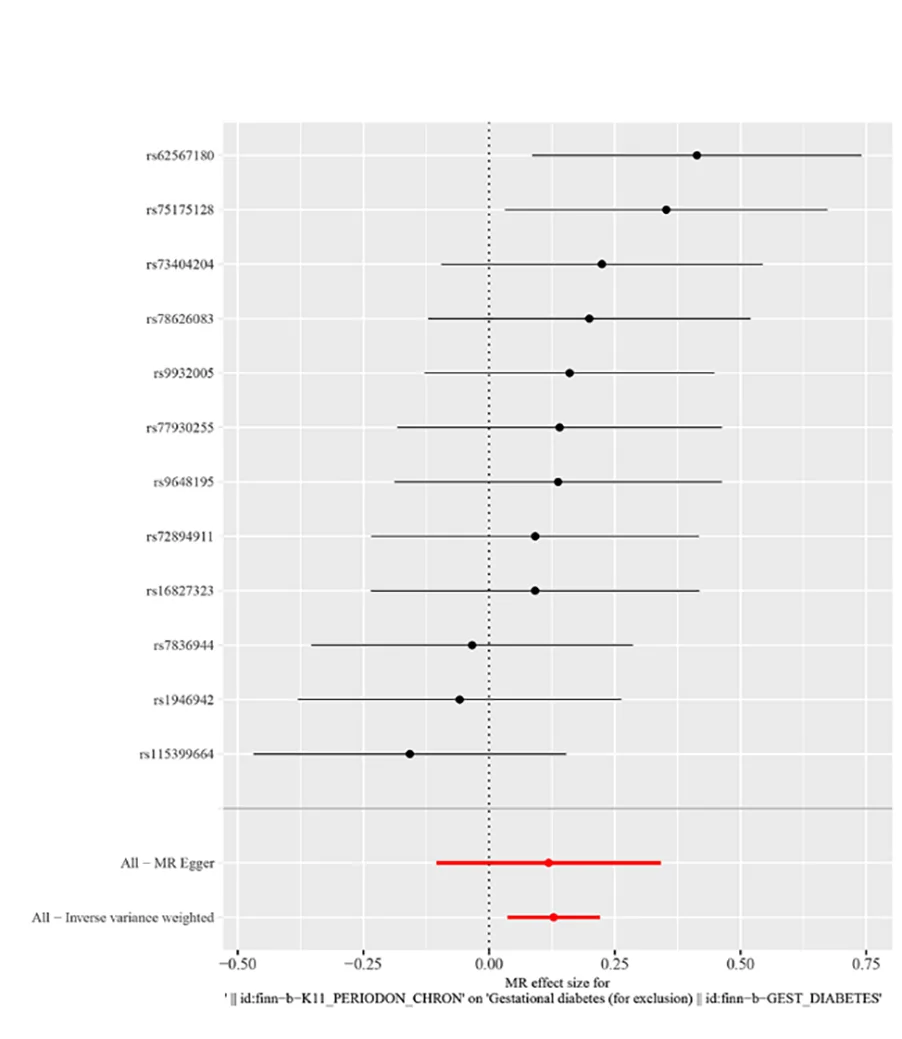

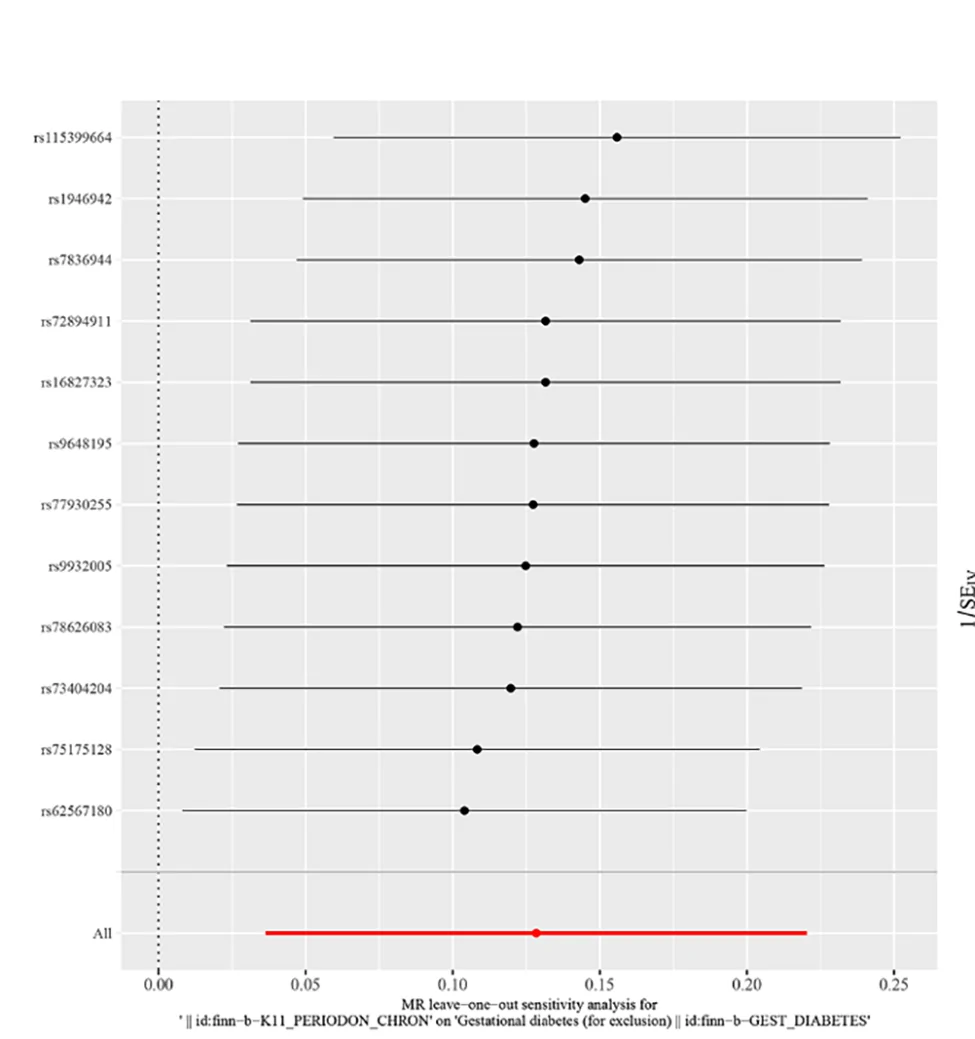

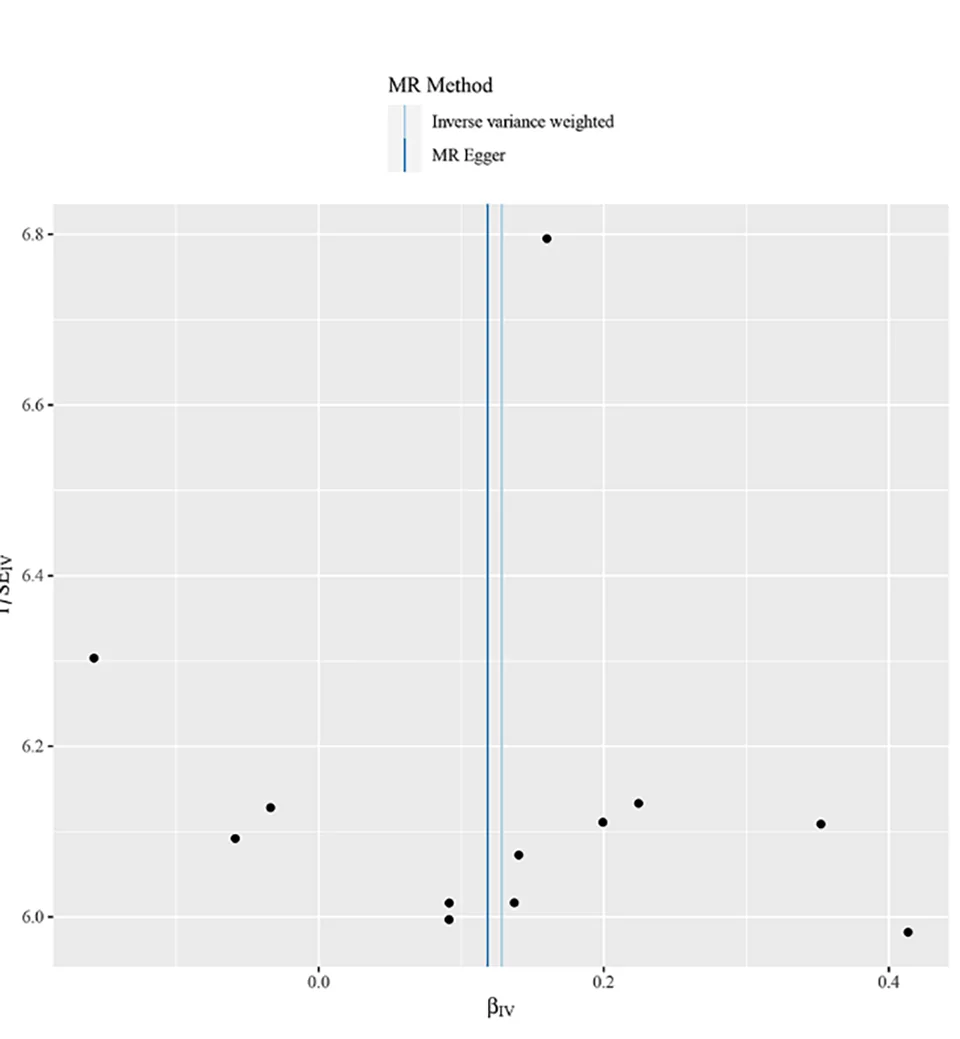
MR results and sensitivity analyses for the effect of PD on GDM. Scatter plot of SNP effects on PD (x-axis) and SNP effects on GDM (y-axis) under five different MR methods. Each coloured line corresponds to a different method and its slope represents the estimated MR association between genetically predicted PD and GDM risk (a positive slope indicates an increased GDM risk due to PD, whereas a negative slope would indicate a protective effect) (a). Forest plot of causal effect estimates for PD on GDM derived from individual SNPs (Wald ratios). Each horizontal line indicates the effect size and confidence interval (CI) for a single SNP. Lines entirely to the right of the zero line suggest that PD increases GDM risk, lines to the left suggest a risk-lowering effect, and lines crossing zero indicate no significant effect (b). Leave-one-out analysis. Each point represents the overall MR estimate (IVW method) after excluding one SNP at a time (error bars show the 95% CI). The consistency of the estimates indicates no single SNP is unduly influencing the result (c). Funnel plot of the SNP-specific MR estimates (effect size vs precision). The approximately symmetric distribution of points suggests the absence of directional pleiotropy (d).

#### Sensitivity analyses

Cochran Q tests (Table 2) for both MR-Egger and IVW models indicated no significant heterogeneity in either the forward or reverse analysis (*P* > 0.05). Leave-one-out analysis showed that for the forward MR, the overall causal estimate was not markedly altered by the removal of any single SNP, suggesting that no individual instrument was unduly driving the effect (Fig 2c). The funnel plot of the SNP-specific estimates was roughly symmetrical (Fig 2d), providing no evidence of strong directional pleiotropy. Furthermore, MR-Egger intercept tests for both the forward and reverse analyses did not detect any significant horizontal pleiotropy (*P* > 0.05), indicating that the MR results are not biased due to pleiotropic effects.

**Table 2 Table2:** Cochran Q test of MR estimates between PD and GDM

Exposure	Outcome	Method	Q statistic	df	*P* value
PD	GDM	MR Egger	1.456	5	0.918
PD	GDM	IVW	2.450	6	0.874
GDM	PD	MR Egger	8.757	14	0.846
GDM	PD	IVW	10.183	15	0.808
df, degrees of freedom; Q, Cochran Q test statistic.					

### Bioinformatics Analysis

#### MR-related candidate genes

A total of six MR-related candidate genes were mapped from MR-associated SNPs: HSPBAP1, NDUFAF6, APBA1, SHC4, DGKB, and CDH1 (Table 3). Several mapped SNPs were located in intronic or intergenic regions; therefore, these genes should be interpreted as nearby or annotated candidate genes rather than causal genes. Three of them were present in the PD dataset and five in the GDM dataset. The expression heatmap (Fig 3a) indicated that the MR-genes showed significant co-expression correlations in the GDM samples, whereas no strong correlations were observed in PD. Figure 3b illustrates the differences in MR-related candidate genes between cases and controls. CDH1 expression was significantly higher in GDM patients compared to controls. In the correlation analysis (Fig 3c), the expression of HSPBAP1 was correlated with NDUFAF6 (R = 0.91), APBA1 was correlated with SHC4 (R = 0.81), and APBA1 was correlated with NDUFAF6 (R = 0.75). Additionally, CDH1 expression was positively correlated with HSPBAP1 in both the PD and GDM datasets.

**Table 3 Table3:** MR-gene results associated with exposure (PD) and outcome (GDM)

SNP	Type	Loc hg19	Alleles	Gene
rs115399664	Downstream gene variant	3:122458770	C>T	HSPBAP1
rs16827323	Intergenic variant	3:117325738	C>T	NA
rs1946942	Intron variant, non-coding transcript variant	8:96102796	C>T	NDUFAF6
rs62567180	Intron variant	9:72096659	C>A,T	APBA1
rs72894911	Intergenic variant	6:80481546	A>C	NA
rs73404204	Intron variant	15:49250265	A>G	SHC4
rs75175128	Intergenic variant	6:145696377	A>G	NA
rs77930255	Intergenic variant	1:163913563	C>A,T	NA
rs7836944	Intergenic variant	8:135436202	G>A	NA
rs78626083	Intergenic variant	5:104985928	A>G	NA
rs9648195	Intron variant	7:15012390	C>A,T	DGKB
rs9932005	Intron variant	16:68822019	C>A,G,T	CDH1
NA, not applicable.				

**Fig 3a to c Fig3atoc:**
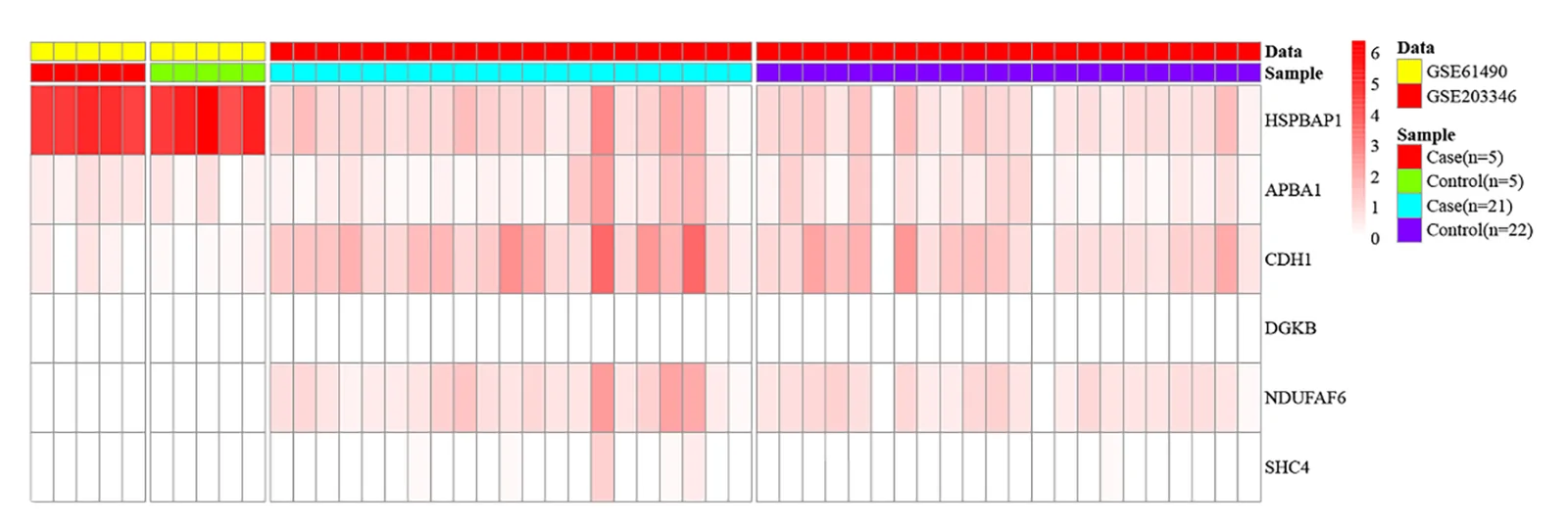

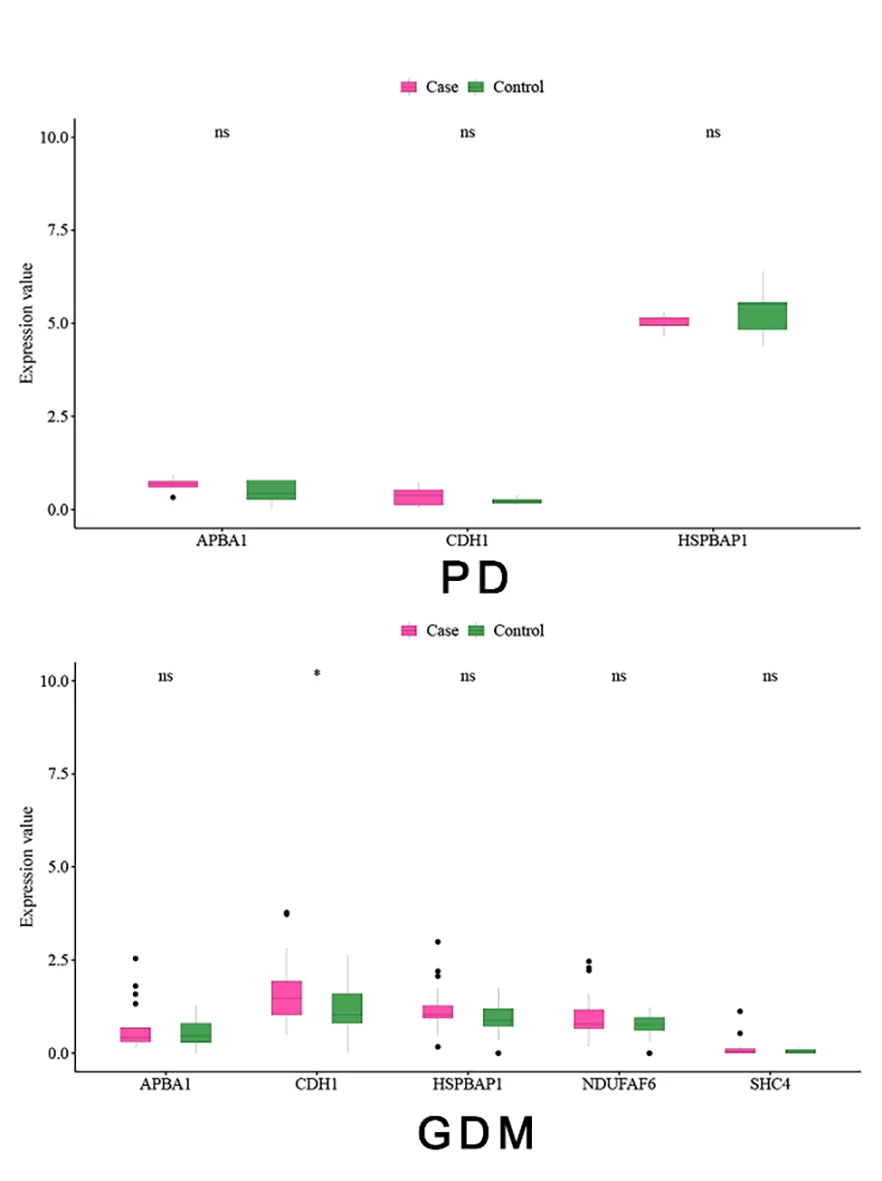

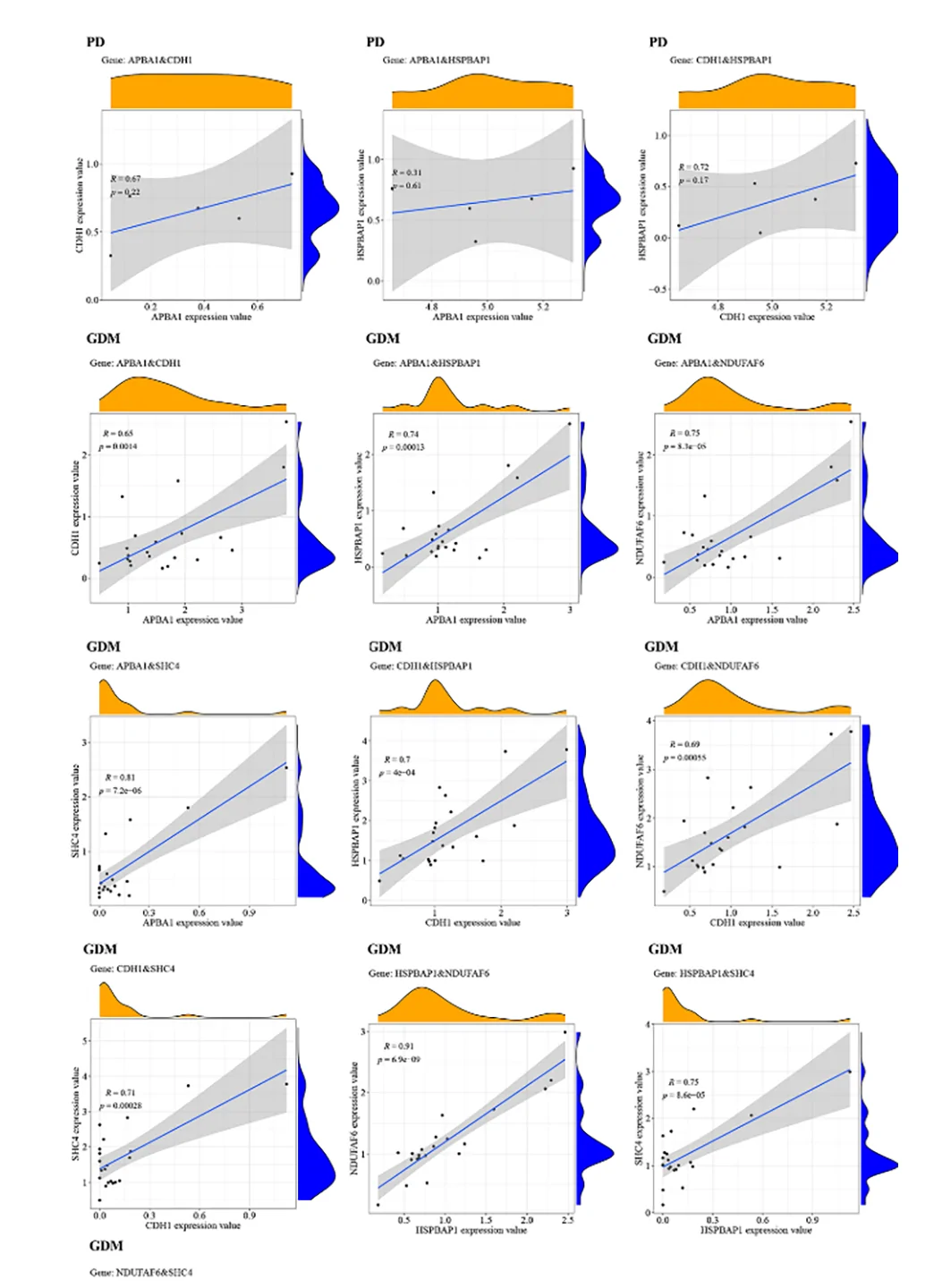
MR-genes expression differences and correlations in PD and GDM. Heatmap of MR-gene expression in the PD (GSE61490) and GDM (GSE203346) datasets (a); differential expression of MR-genes in PD and GDM cases vs controls (boxplots) (b); correlation matrix among MR-genes in PD and GDM (c). ns, non-significant; **P* < 0.05.

#### PD-DEGs and GDM-DEGs

There were 158 overlapping genes identified between the PD- and GDM-related gene sets (Fig 4a). Using the exploratory threshold described above, 10 candidate PD-DEGs and 6 candidate GDM-DEGs were retained for downstream analyses (Table 4). Figure 4b shows the expression of these DEGs and Fig 4c presents the correlation relationships among them. In the PD, CDH1 was positively correlated with HLA-DRB1 (R = 0.94); APBA1 was negatively correlated with GPX1 (R = −0.89); and GPX1 was negatively correlated with HLA-DRB1 (R = −0.73). In the GDM, HSPBAP1 and APBA1 both showed positive correlations with NOS3 (R = 0.89, 0.86), and NOS3 was positively correlated with GATA3 (R = 0.92) and AKT1 (R = 0.86).

**Fig 4a to c Fig4atoc:**
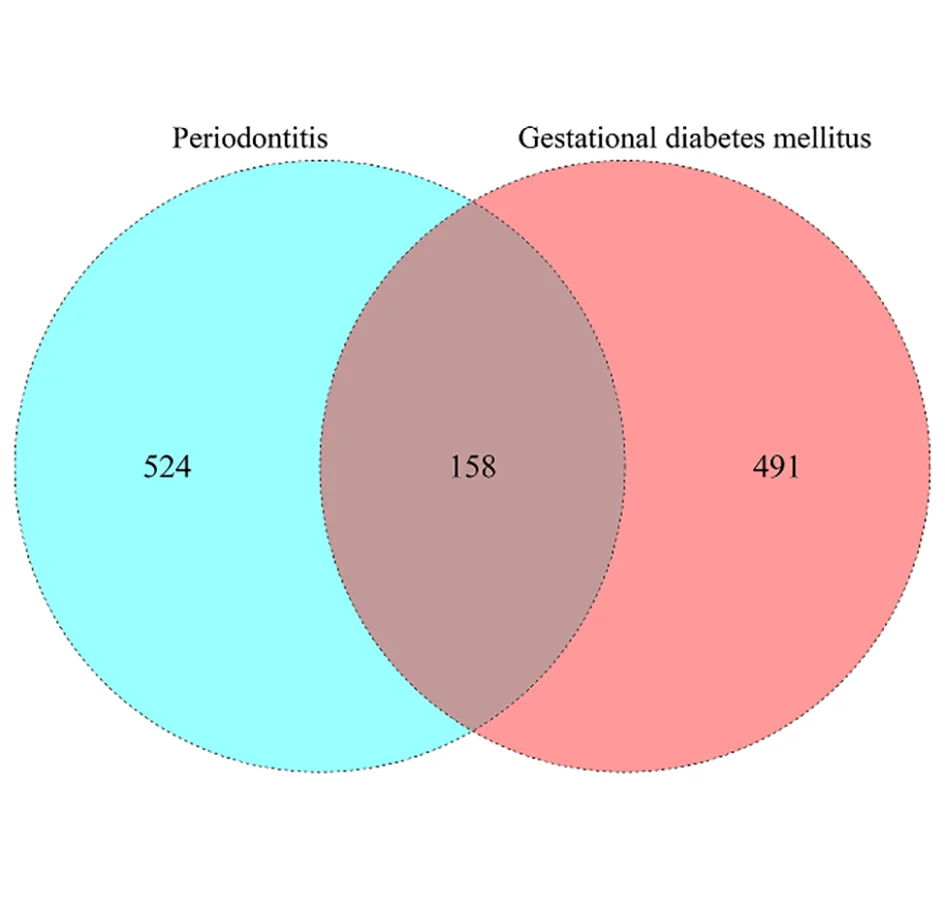

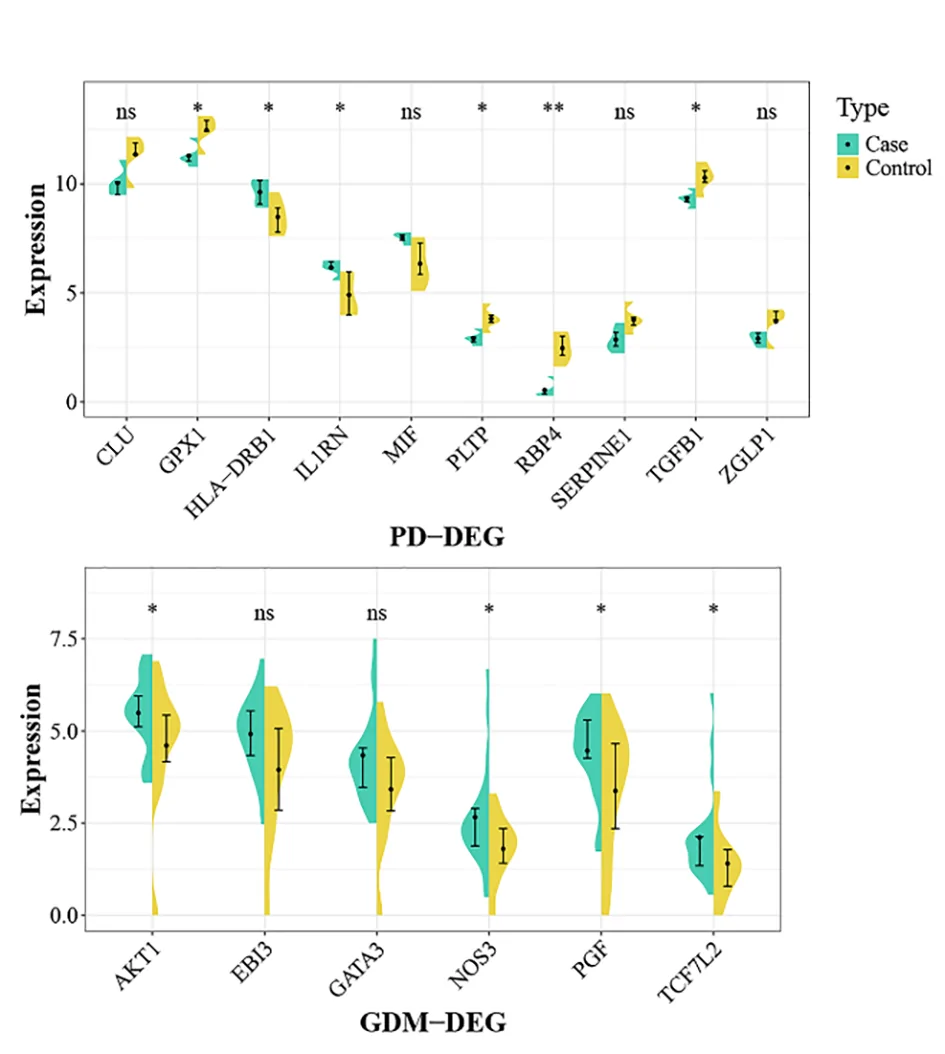

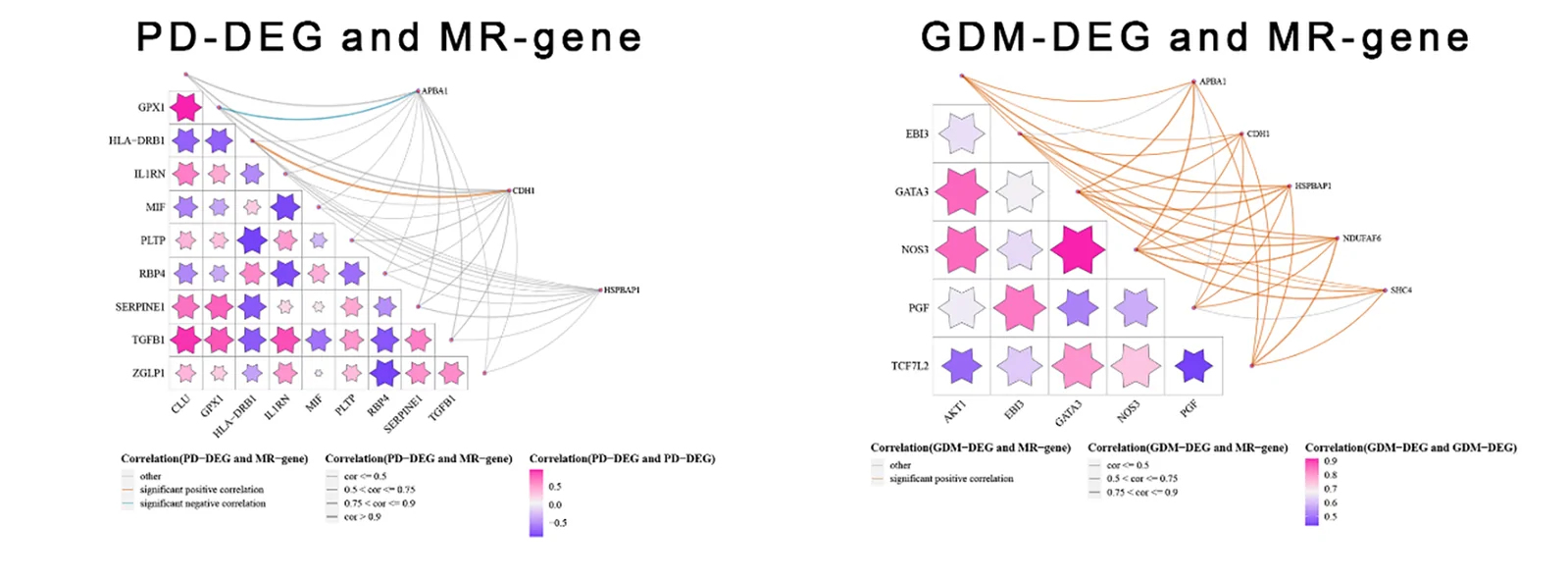
Overlapping DEGs in PD and GDM and the expression correlations. Venn diagram of shared genes between periodontitis-associated and GDM-associated gene sets (a); selected differentially expressed genes in PD and GDM datasets (b); pairwise gene correlation analysis for the identified DEGs in PD and GDM (c).

**Table 4 Table4:** Differential expression analysis result between PD and GDM

Group	Gene	Log FC	*P* value	Regulate
PD-DEG	HLA-DRB1	1.143	0.018	Up
	IL1RN	1.255	0.014	Up
	MIF	1.197	0.018	Up
	CLU	−1.312	0.014	Down
	GPX1	−1.179	0.007	Down
	PLTP	−0.897	0.005	Down
	RBP4	−1.924	< 0.001	Down
	SERPINE1	−0.905	0.019	Down
	TGFB1	−0.956	0.009	Down
	ZGLP1	−0.785	0.038	Down
GDM-DEG	PGF	1.092	0.017	Up
	NOS3	0.857	0.020	Up
	EBI3	0.971	0.030	Up
	GATA3	0.920	0.032	Up
	TCF7L2	0.713	0.039	Up
	AKT1	0.883	0.047	Up
DEG, differentially expressed gene; FC, fold change.				

#### Crosstalk genes

Combining 22 genes yielded the crosstalk gene set. Enrichment analysis (Fig 5a) revealed that the crosstalk genes were primarily involved in the regulation of apoptotic signalling pathways, regulation of cell-cell adhesion, and lipopolysaccharide-mediated signalling. They were also significantly enriched in several relevant pathways, including the AGE–RAGE signalling pathway in diabetic complications, Th17 cell differentiation, the relaxin signaling pathway, and the HIF-1 signalling pathway. These exploratory findings suggest that apoptosis, inflammation, immune responses, and hormonal signalling may represent candidate biological processes linking PD and GDM.

**Fig 5a to c Fig5atoc:**
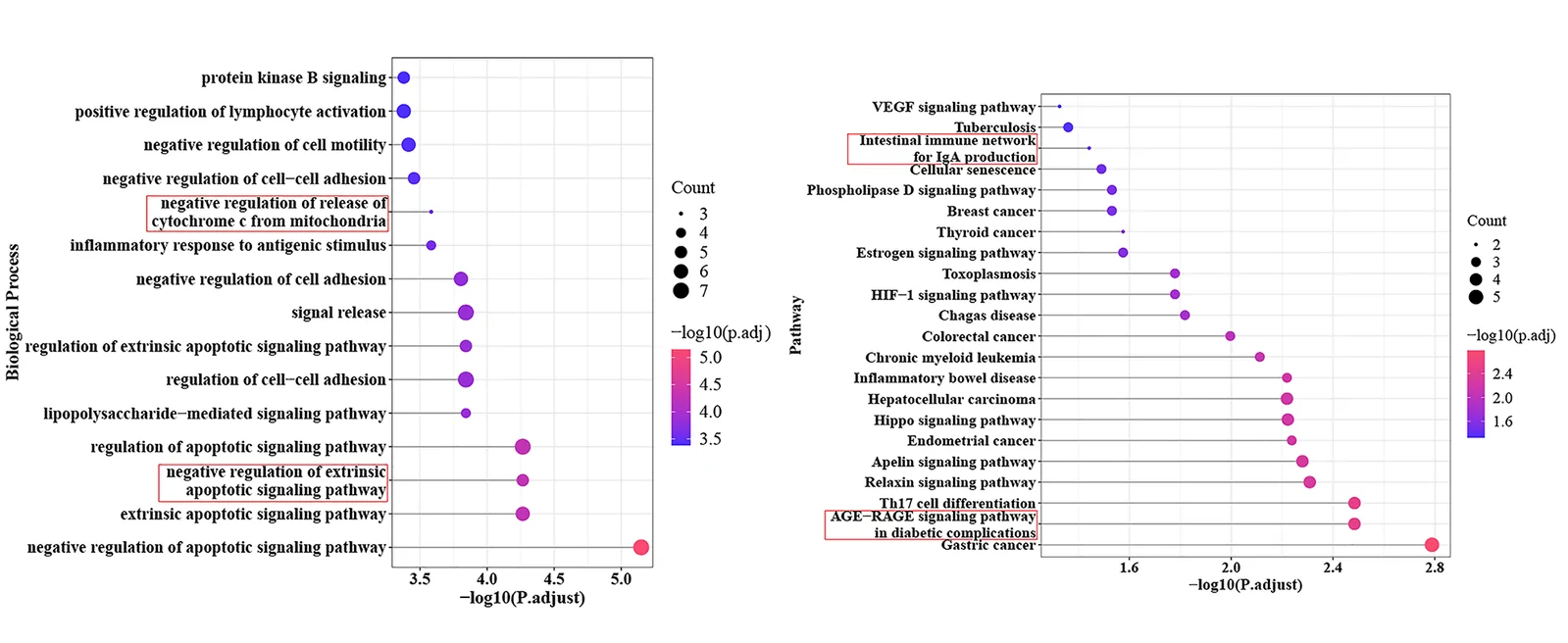

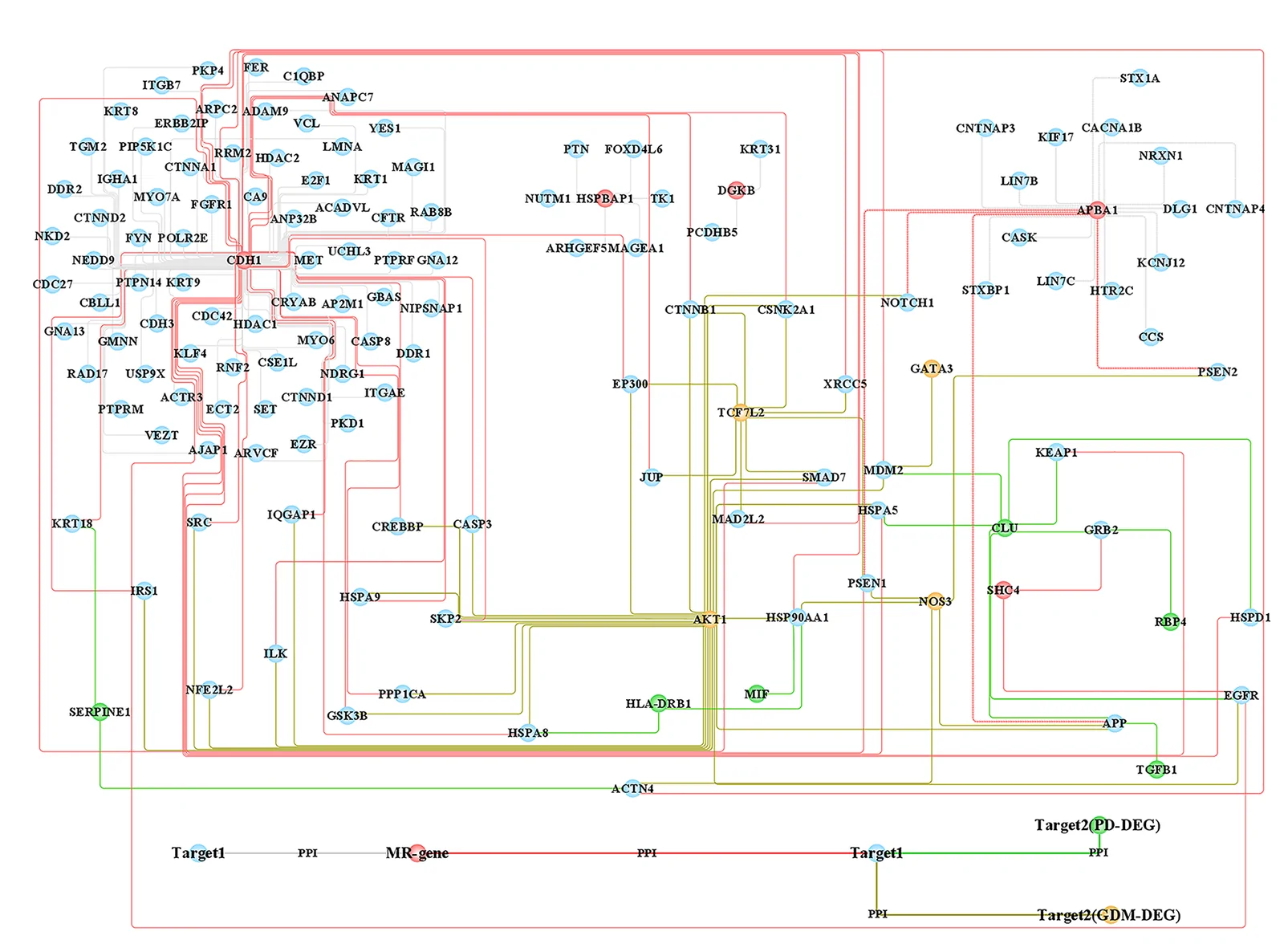

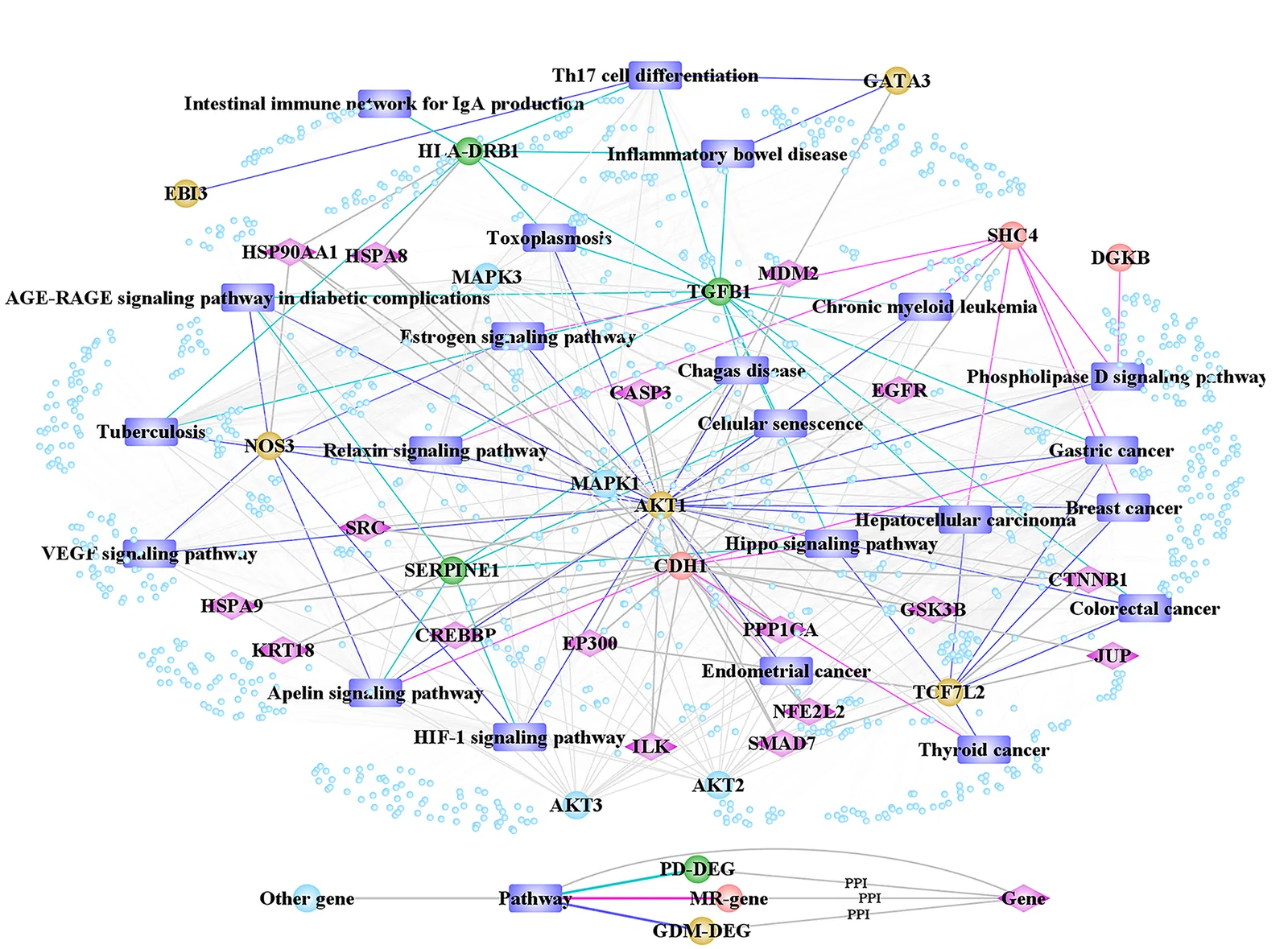
Exploratory network analysis of candidate genes potentially linking PD and GDM. Top enriched Gene Ontology (GO) biological processes and Kyoto Encyclopedia of Genes and Genomes (KEGG) pathways (a); protein-protein interaction (PPI) network showing possible connections between MR-related candidate genes and candidate PD-/GDM-DEGs through first- and second-order interacting partners (b); pathway–gene map showing candidate genes mapped to pathways of interest (c). These network analyses are intended to visualise possible biological relationships and should be interpreted as hypothesis-generating rather than confirmatory.

#### PPI network construction

Five out of the six MR-genes (CDH1, HSPBAP1, APBA1, DGKB, and SHC4) had reported protein interaction partners in the queried databases. The constructed MR-gene–Target1–Target2 network (Fig 5b) revealed connections between the MR-genes and the dysregulated genes from PD and GDM. Specifically, six PD-DEGs (CLU, HLA-DRB1, MIF, RBP4, SERPINE1, and TGFB1) and four GDM-DEGs (AKT1, GATA3, NOS3, and TCF7L2) were identified within the network. MR-related candidate genes such as CDH1, APBA1, and SHC4 were connected to candidate DEGs through bridging proteins in the exploratory PPI network, suggesting possible indirect relationships that warrant further functional validation.

#### Pathway–gene crosstalk analysis

Figure 5c illustrates which significant KEGG pathways involve the crosstalk genes. Pathway mapping suggested that MR-related candidate genes such as CDH1 and SHC4 may be associated with several pathways of interest, including Apelin, Hippo, estrogen, relaxin, and phospholipase D signalling pathways. Additionally, several important DEGs were mapped to these pathways: AKT1 (GDM-DEG), NOS3 (GDM-DEG), and SERPINE1 (PD-DEG) were all part of the Apelin signalling pathway, and AKT1, NOS3, and SERPINE1 (PD-DEG) were involved in the estrogen signalling pathway. Through interactions identified in the PPI network, CDH1 could indirectly associate with AKT1 via the intermediary ILK protein, highlighting a link between cell adhesion pathways and insulin signalling in the context of PD and GDM.

### Exploratory In Vitro Assessment

#### Validation of protein and mRNA expression by WB and qRT-PCR

qRT-PCR analysis (Fig 6b, d and f) showed that, compared with the GDM group, the GDM + PD group had increased mRNA expression of HSPBAP1, APBA1, and SHC4 in MR-genes, as well as elevated mRNA levels of all inflammatory cytokines and oxidative stress–related genes. In contrast, the mRNA levels of NDUFAF6, DGKB, and CDH1 were significantly lower in the GDM +PD group than in the GDM group. WB results (Fig 6a, c and e) further confirmed the qRT-PCR findings, with protein expression changes mirroring the mRNA results (Supplementary Table 1). The statistical significance of these expression differences varied across markers.

**Fig 6a to g Fig6atog:**
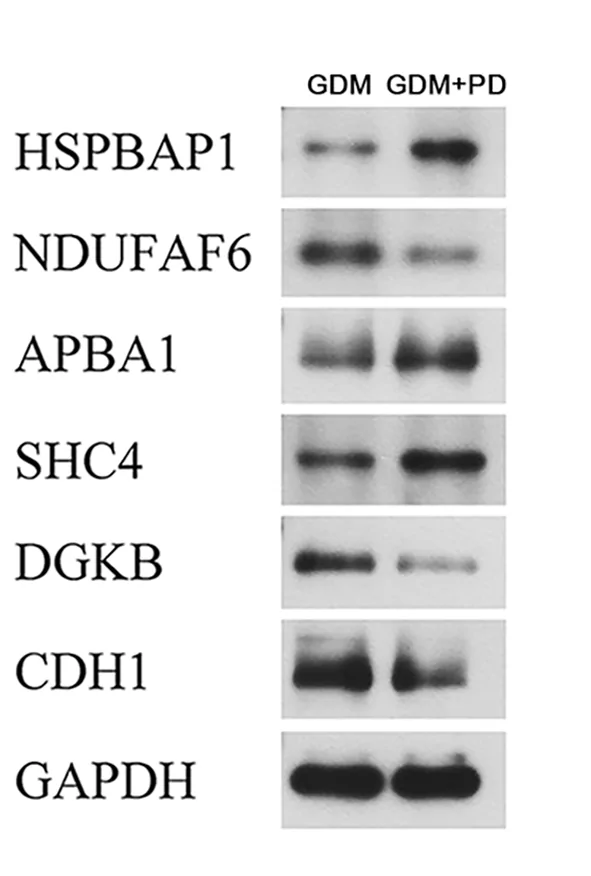

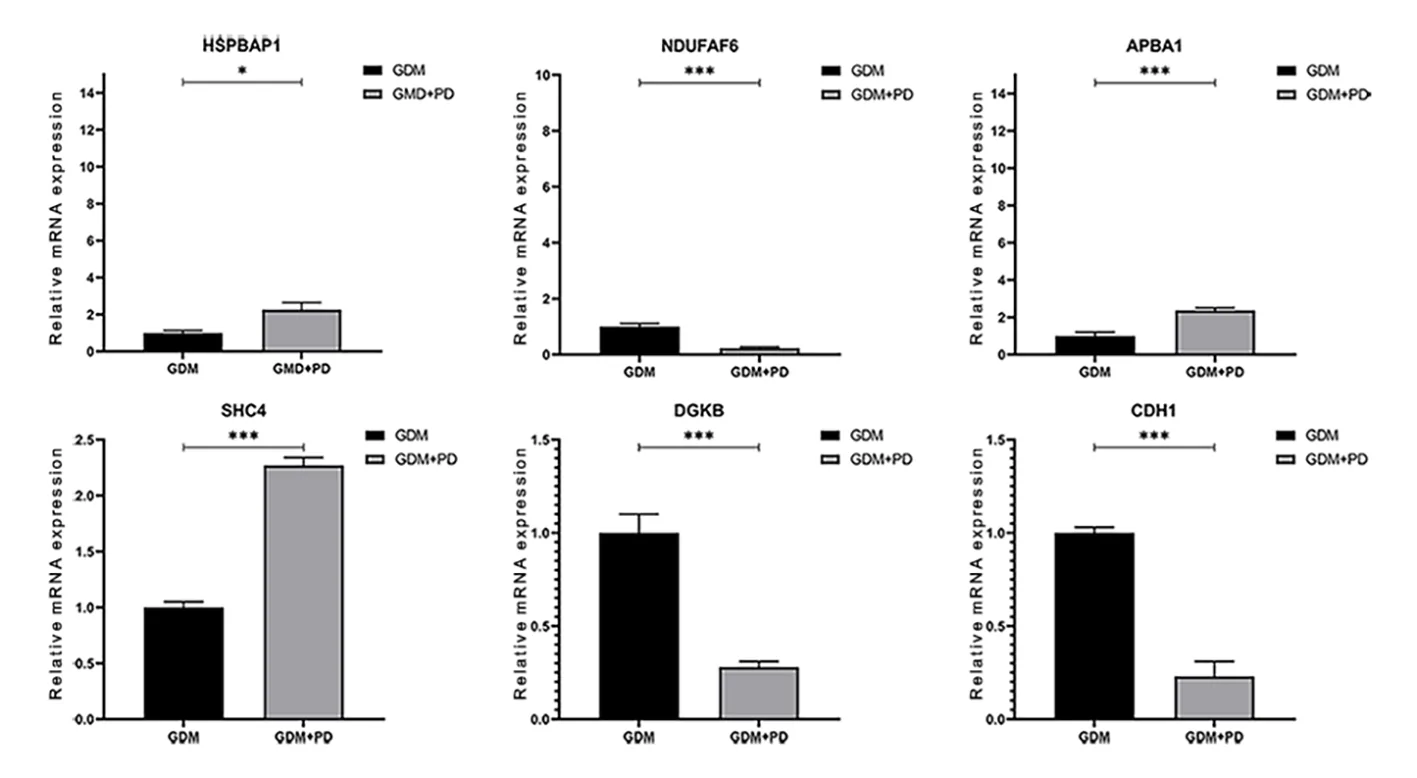

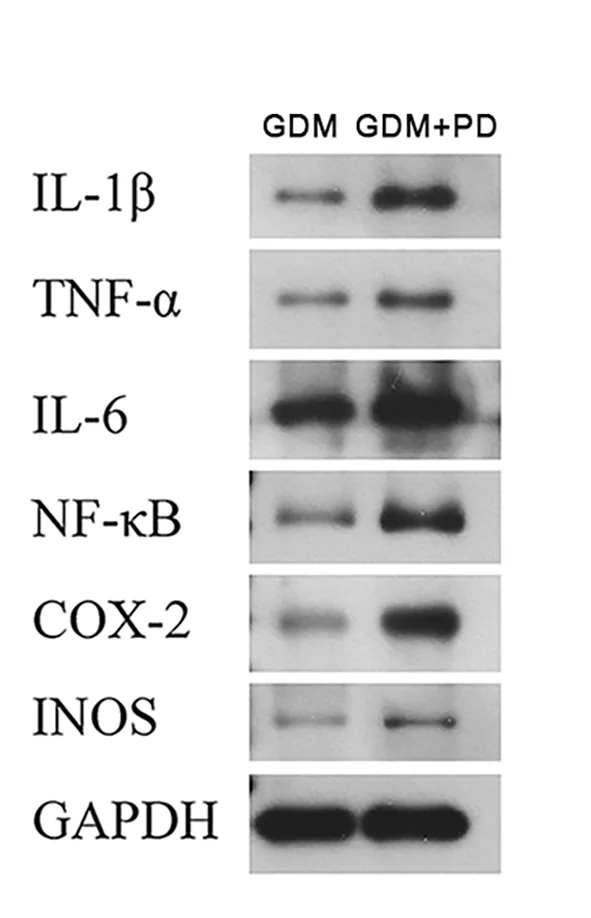

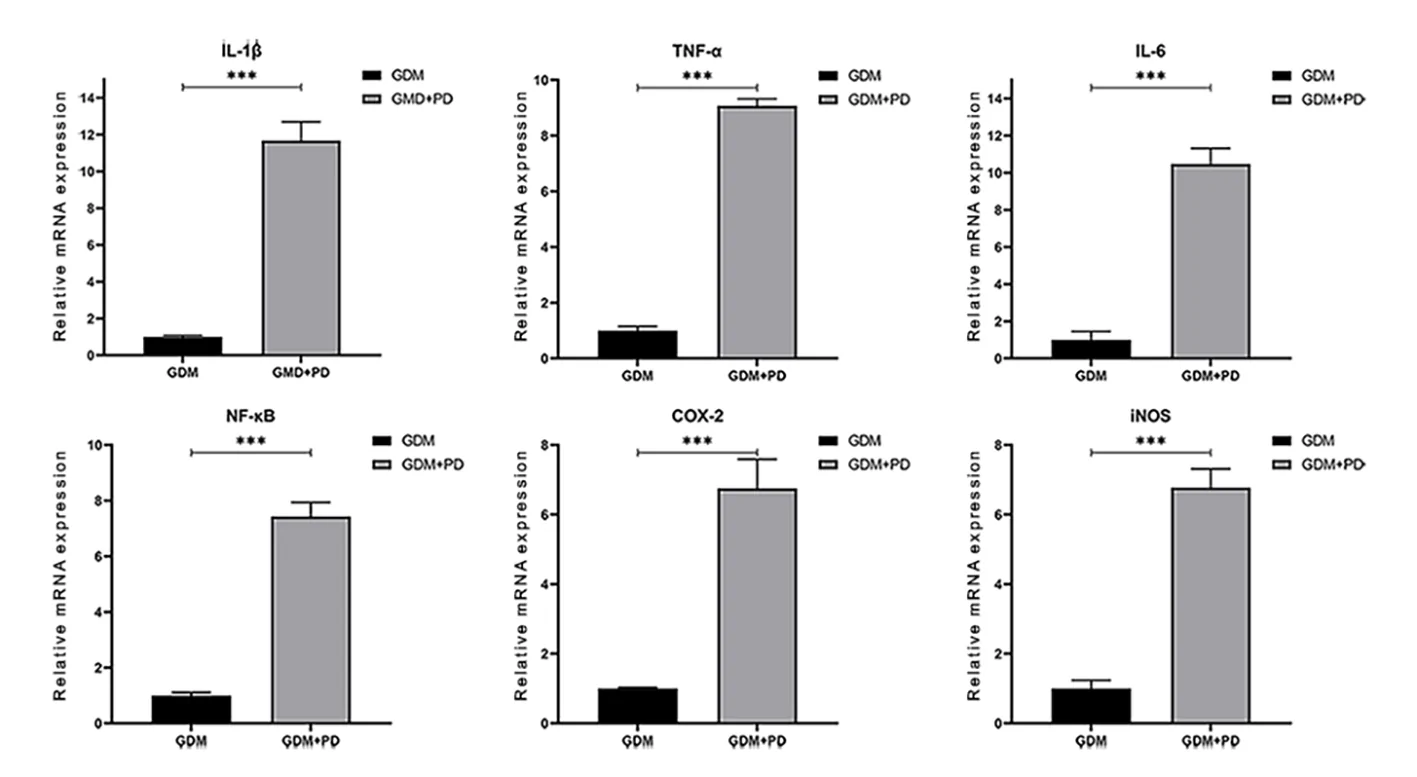

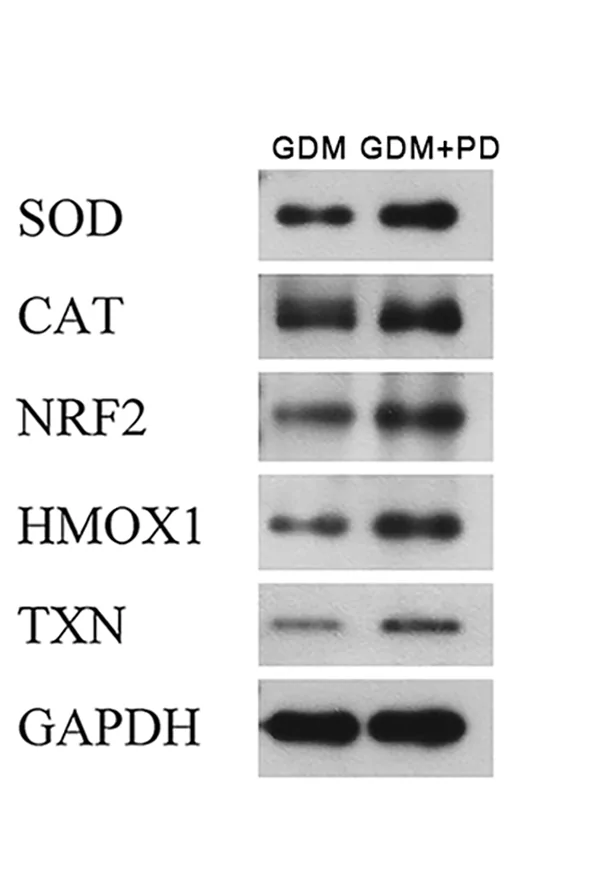

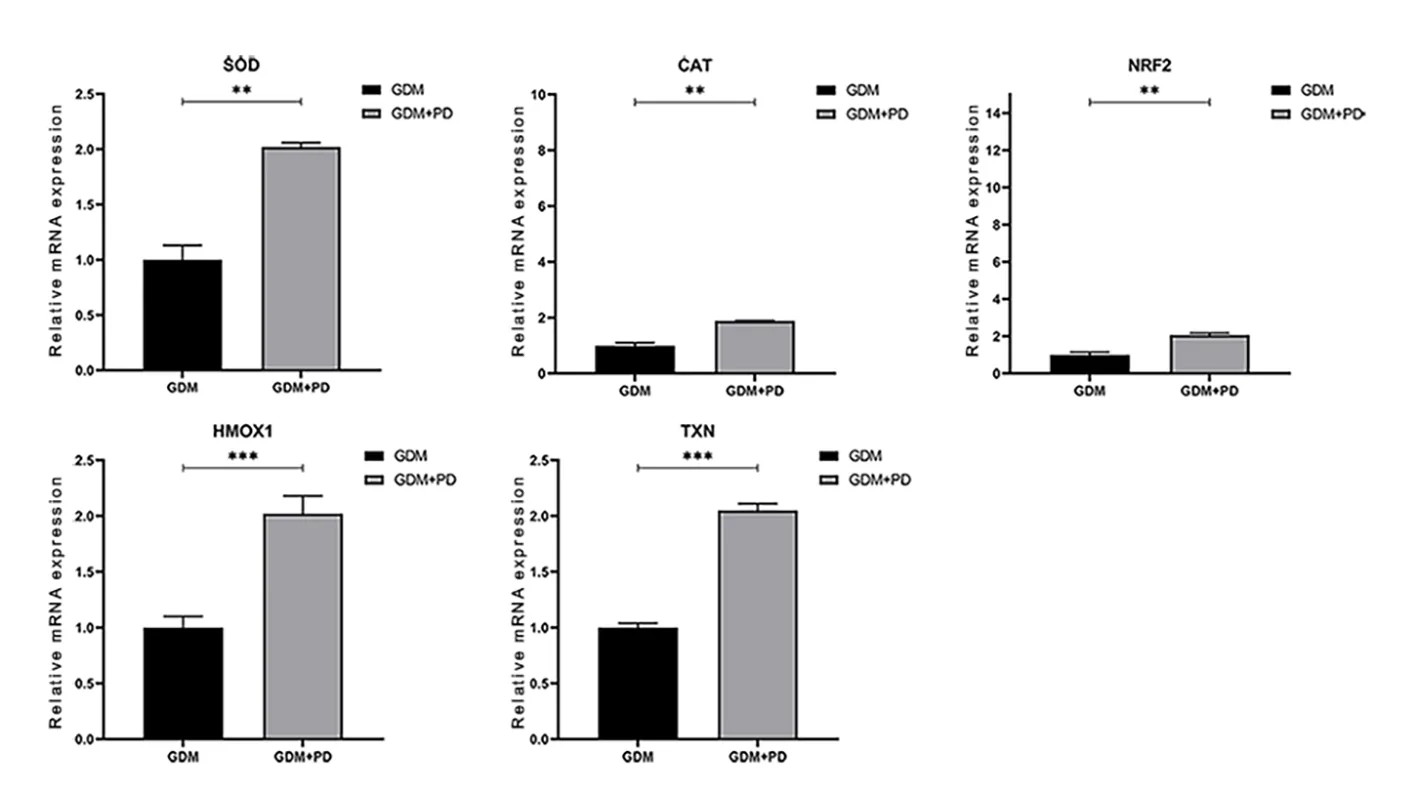

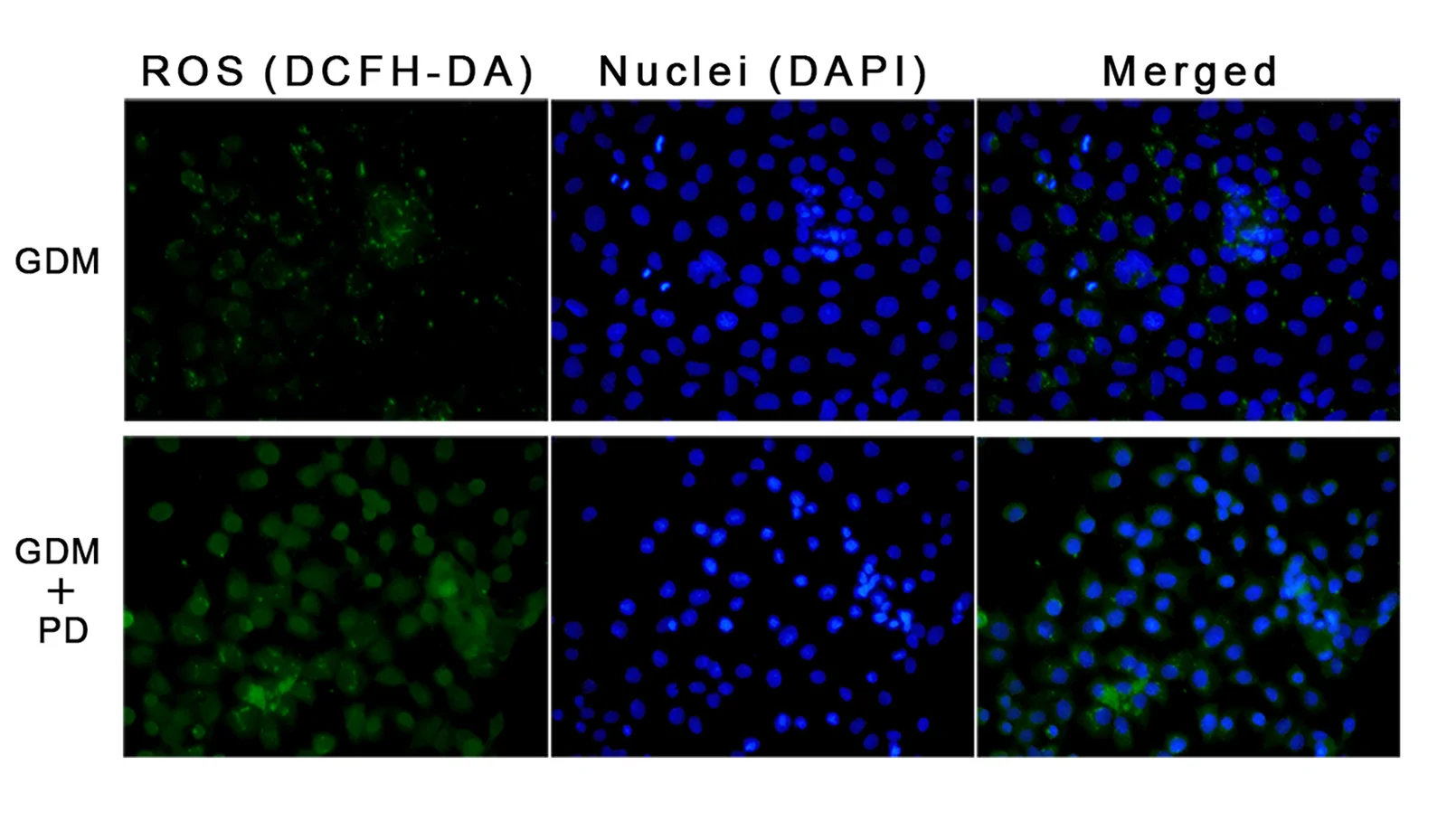
Experimental validation of the PD–GDM interaction. WB results for MR-gene proteins (a); qRT-PCR for MR-genes (b); WB results for of inflammatory cytokines (c); qRT-PCR results for inflammatory cytokines (d); WB results for oxidative stress (e); qRT-PCR results for oxidative stress (f); fluorescence microscopy images (10 × 40) of ROS (g). **P* < 0.05, ***P* < 0.01, ****P* < 0.001.

#### Assessment of oxidative stress by ROS

Figure 6g shows the fluorescence-based detection of ROS in the EndoC-βH1 cells under the two conditions. In the GDM group, the cells exhibited only faint green fluorescence, with a sparse and diffuse distribution, indicating low baseline ROS levels. In contrast, the GDM + PD group showed markedly stronger green fluorescence intensity throughout the cells, indicating a significant elevation in intracellular ROS levels when Pg was present. This suggests that the addition of the periodontal pathogen under high-glucose, high-hormone conditions substantially increased oxidative stress in the β-cells. In both groups, the cell nuclei (stained blue with DAPI) were clearly visible and intact, and overlaying the images confirmed that the ROS-associated fluorescence was primarily localised in the cytoplasm.

## Discussion

This study provides multilevel evidence supporting a potential biological link between PD and GDM. The forward MR analysis showed evidence consistent with a modest positive association between genetically predicted PD and GDM risk, whereas the reverse analysis did not provide clear evidence for an association between genetically predicted GDM and PD risk. Together with the exploratory bioinformatics analyses and simplified in vitro β-cell challenge experiments, these findings suggest that inflammatory responses and oxidative stress may be involved in the PD-GDM link. However, given the modest effect size and the use of a relaxed instrumental variable threshold, these findings should be interpreted as supportive rather than definitive evidence of causality.

Unlike the well-established bidirectional relationship between diabetes and PD,^33^ this study was more consistent with a potential directional association from PD to GDM than with the reverse direction. However, the magnitude of the association was modest, with the IVW estimate corresponding to an approximately 14% higher odds of GDM. This emphasises the unique importance of oral health management in pregnant women. These findings provide a rationale for future prospective and interventional studies to examine whether improving periodontal health before or during early pregnancy may influence inflammatory markers, glycaemic regulation, or GDM risk.^17,31
^ Recent periodontal literature also supports this cautious perspective by emphasising that periodontal disease may have systemic implications and that adjunctive antimicrobial or host-modulatory approaches in nonsurgical periodontal therapy may be particularly relevant for metabolically susceptible populations, including patients with diabetes.^10,36
^ It is noteworthy, however, that the reverse MR analysis did not provide clear evidence supporting an effect of genetically predicted GDM on PD risk. One possible explanation is that GDM is a transient condition limited to the duration of pregnancy, and the period of hyperglycaemia may be too short to cause substantial structural damage to periodontal tissues within that timeframe, making a measurable causal effect harder to detect. Nevertheless, this does not imply that GDM has no influence on periodontal health at all. Existing evidence suggests that even short-term hyperglycaemia can alter immune responses and increase inflammatory cytokine levels,^11^ which could adversely affect periodontal tissues under certain conditions. For example, in the presence of obesity or insulin resistance, the impact of hyperglycaemia on the periodontium might be more pronounced.^7,12
^ Another consideration is that advancing maternal age and repeated GDM across multiple pregnancies may amplify the cumulative effect of hyperglycaemia on periodontal health. Older women experiencing GDM in successive pregnancies may face prolonged or repeated exposure to inflammatory stress, potentially increasing the risk of periodontal deterioration beyond what occurs in a single pregnancy.^24,26,34
^ Therefore, further research with larger sample sizes, prospective designs, and stratification by gestational timing is warranted to investigate any potential impact of GDM on PD.

Through exploratory transcriptomic differential expression analysis, this study prioritised several candidate genes that may be involved in both PD- and GDM-related biological processes, including inflammatory mediators and metabolism-related genes. Many of these genes are directly involved in inflammation or in the regulation of pancreatic β-cell function and insulin signalling, suggesting that periodontitis might contribute to GDM by intensifying systemic inflammation and disrupting metabolic homeostasis in islet cells. The in vitro experimental validation also supports this hypothesis. Significant upregulation of inflammatory cytokines and oxidative stress markers was observed, coupled with dysregulation of key MR-genes. Specifically, genes involved in β-cell function (DGKB) and cell adhesion (CDH1) were downregulated, while stress-response genes (HSPBAP1) and inflammatory pathway components were upregulated in the GDM+PD group compared to GDM alone. The presence of Pg dramatically increased intracellular ROS levels, demonstrating that periodontal bacteria can directly induce oxidative stress in β-cells beyond the effects of hyperglycaemia and hormonal changes. This oxidative burden, combined with inflammatory activation, likely contributes to β-cell dysfunction and impaired insulin secretion, characteristic of GDM. The functional enrichment analysis provided hypothesis-generating support for this possibility: the crosstalk genes were significantly enriched in pathways related to diabetic complications (AGE–RAGE signalling), immune responses (Th17 cell differentiation), hypoxia and stress responses (HIF-1 signalling), and pregnancy hormones (estrogen and relaxin signalling). For instance, excessive accumulation of advanced glycation end-products (AGEs) can bind to the RAGE receptor and activate downstream NF-κB signalling, leading to overproduction of TNF-α, IL-1β, and other cytokines that exacerbate tissue inflammation and damage.^29^ Th17-mediated immunity, known to be upregulated in periodontitis, plays a central role in sustaining periodontal inflammation and could contribute to insulin resistance when amplified systemically, thereby linking PD to GDM pathogenesis.^16^ Besides, the PPI network observed that certain MR-genes (CDH1, APBA1, SHC4) were connected to inflammatory factors through bridging proteins, forming an interaction network that intertwines inflammatory pathways with cell adhesion and metabolic signalling pathways. This implies that periodontal inflammation may not only elevate systemic pro-inflammatory mediators but also alter the cellular environment of pancreatic islets (for example, by affecting β-cell adhesion and survival), ultimately perturbing maternal glucose regulation. Additionally, the identification of pregnancy-related hormone signalling pathways (estrogen, relaxin) in the analysis suggests that the endocrine changes during gestation might modulate the interplay between PD and glucose metabolism. Chronic periodontal infection could disrupt the local balance of these hormones^25^ and reduce protective hormone levels,^38^ thereby potentially intensifying the adverse metabolic impacts of periodontitis during pregnancy. In summary, the genetic and molecular findings outline a plausible mechanism in which periodontitis-induced inflammation and oxidative stress converge on critical signalling pathways, leading to impaired glycaemic control in pregnancy.

The in vitro experiments provided exploratory biological evidence that Pg exposure may aggravate β-cell inflammatory and oxidative stress responses under high-glucose and progesterone conditions. Under high-glucose and high-hormone (progesterone) conditions akin to GDM, the addition of the periodontal pathogen Pg led to markedly heightened inflammation and oxidative stress in human β-cells. Significant upregulation of pro-inflammatory cytokines (IL-1β, TNF-α, IL-6) and activation of the NF-κB pathway were observed, along with increased expression of inflammatory enzymes like COX-2 and iNOS, in the GDM + PD group compared to high glucose alone. Concurrently, there was a substantial rise in ROS production. These data suggest that periodontal infection can act synergistically with the hyperglycaemic, pro-inflammatory state of GDM to elicit an exaggerated immune-inflammatory response and oxidative injury in β-cells. Besides, the changes in expression of the MR-genes in the GDM + PD group, such as the upregulation of HSPBAP1, APBA1, and SHC4 and the downregulation of CDH1 under combined GDM and PD conditions, mirror the patterns expected from the bioinformatic analyses. This concordance reinforces the idea that periodontitis exacerbates the inflammatory and stress pathways that impair β-cell function. These findings are same with a previous animal studies where chronic oral infection was found to induce pancreatic inflammation and β-cell damage, presumably through bacterial translocation and systemic inflammatory mediators.^18^ Thus, the experimental results provide limited supportive evidence that periodontal bacterial exposure may enhance inflammatory and oxidative stress pathways in β-cells under GDM-like conditions. However, these findings should not be interpreted as demonstrating that periodontitis directly causes GDM.

Overall, the findings provide a potential cross-disciplinary perspective for future research on the relationship between periodontal health and GDM. By combining genetic epidemiology, bioinformatics, and experimental biology, this study has constructed a comprehensive evidence framework that moves from demonstrating a causal link to elucidating molecular mechanisms and then confirming those mechanisms in a biological system. This multistage approach enhances the robustness of the conclusions, providing compelling evidence that PD can causally contribute to GDM and highlighting key pathways for this interaction.

However, this study has some limitations. First, the MR analysis was based on GWAS data predominantly from European-ancestry populations. Genetic and environmental differences in other populations may affect the relationship between PD and GDM; thus, the findings should be validated in more diverse cohorts. Second, the MR findings should be interpreted cautiously because the instrumental variables were selected using a relaxed significance threshold of *P* < 5 × 10^-6^ rather than the conventional genome-wide significance threshold. Although this strategy increased the number of available instruments for this exploratory MR analysis, it may also increase susceptibility to weak instrument bias or residual pleiotropy. In addition, the number of instruments was relatively small, particularly in the forward analysis, which limited the statistical power of pleiotropy-robust methods such as MR-Egger regression and reduced the ability to detect subtle horizontal pleiotropy. Although no strong evidence of heterogeneity or directional pleiotropy was detected in the sensitivity analyses, these tests cannot completely exclude pleiotropic pathways. The MR findings should therefore be regarded as supportive evidence consistent with a potential effect rather than definitive proof of causality. In addition, mapping MR-associated SNPs to nearby genes provides candidate loci only and does not establish that these genes are causal mediators of the PD–GDM association. Third, while the in vitro β-cell model probed specific mechanisms, it cannot fully simulate the complex immunological and hormonal environment of an actual pregnancy. The interactions between maternal immune adaptation, endocrine fluctuations, and oral pathogens are multifactorial, and further validation using animal models or clinical samples is necessary. Specifically, the direct co-culture of Pg with pancreatic β-cells only represents a simplified model of periodontitis-associated bacteremia. While this model incorporated high glucose and progesterone to simulate GDM conditions, the dynamic fluctuations in glucose levels and hormonal milieu throughout pregnancy are not fully represented. Despite these limitations, the model provides valuable mechanistic insights into how Pg may directly affect β-cell function under GDM-like conditions. In addition, no separate cell viability or cytotoxicity assay was performed under the same exposure conditions, which limits the ability to distinguish specific inflammatory or oxidative stress responses from potential nonspecific cytotoxic effects. Finally, this work represents an initial exploration of the PD-GDM relationship. Future research should include large-scale prospective studies and randomised controlled trials to examine whether improving periodontal health before or during pregnancy influences GDM incidence or severity, inflammatory markers and glycaemic control. Such studies will be crucial to translate the present findings into clinical practice and public health strategies.

## Conclusion

This study provides evidence consistent with a potential biological link between PD and GDM. The MR analysis suggested a modest positive association between genetically predicted PD and GDM risk, while the exploratory bioinformatics analyses and simplified in vitro experiments indicated that inflammatory responses and oxidative stress may be involved. These findings should be interpreted as supportive rather than definitive and warrant further validation in prospective clinical and interventional studies.

This research was supported by the China Scholarship Council (CSC) (CSC no. 202408440099) through a scholarship awarded to Linxin Jiang for doctoral study at Leipzig University.

## References

### Appendix

**Table A1 TableA1:** Grey value and relative expression of protein (normalised to GAPDH)

Sample	Protein	GDM group		GDM + PD group		*P* value	Fold change
		Grey value	Relative expression	Grey value	Relative expression		
Inflammatory cytokines	IL-1β	1084.297	0.1186	3850.816	0.4035	0.030	3.40
	TNF-α	868.8197	0.0950	2073.331	0.2170	0.040	2.29
	IL-6	5621.925	0.6150	8959.385	0.9394	0.061	1.53
	NF-κB	1393.792	0.1527	4365.421	0.4579	0.010	3.00
	COX-2	1006.742	0.1100	4640.803	0.4864	0.020	4.42
	INOS	641.5699	0.0702	1426.078	0.1497	0.049	2.13
	GAPDH	9145.148	1.0000	9549.271	1.0000	NA	NA
MR-genes	HSPBAP1	972.9148	0.1218	3373.158	0.4348	0.030	3.57
	NDUFAF6	3355.175	0.4202	1114.713	0.1436	0.050	0.34
	APBA1	2341.578	0.2930	4444.225	0.5725	0.072	1.95
	SHC4	2775.299	0.3471	4731.05	0.6090	0.060	1.76
	DGKB	3049.547	0.3813	584.7629	0.0754	0.030	0.20
	CDH1	6382.745	0.7984	3617.714	0.4666	0.051	0.58
	GAPDH	7993.604	1.0000	7757.643	1.0000	NA	NA
Oxidative stress-related genes	SOD	3188.042	0.3447	5428.238	0.5324	0.048	1.55
	CAT	5531.546	0.5992	6907.669	0.6789	0.080	1.13
	NRF2	2903.577	0.3144	5858.7	0.5741	0.010	1.83
	HMOX1	1907.847	0.2067	4801.271	0.4936	0.020	2.39
	TXN	986.5293	0.1067	2071.454	0.2059	0.044	1.93
	GAPDH	9244.95	1.0000	10204.47	1.0000	NA	NA
The grey value for the target protein is divided by the grey value of GAPDH, the loading control protein, to calculate the relative expression. The *P* values represent the statistical significance of the difference in relative expression between the groups, calculated using a Student *t* test. The fold change is calculated as the ratio of the relative expression in the GDM + PD group divided by the relative expression in the GDM group. NA, not applicable.							
